# Inactivation of nuclear histone deacetylases by EP300 disrupts the MiCEE complex in idiopathic pulmonary fibrosis

**DOI:** 10.1038/s41467-019-10066-7

**Published:** 2019-05-20

**Authors:** Karla Rubio, Indrabahadur Singh, Stephanie Dobersch, Pouya Sarvari, Stefan Günther, Julio Cordero, Aditi Mehta, Lukasz Wujak, Hector Cabrera-Fuentes, Cho-Ming Chao, Peter Braubach, Saverio Bellusci, Werner Seeger, Andreas Günther, Klaus T. Preissner, Malgorzata Wygrecka, Rajkumar Savai, Dulce Papy-Garcia, Gergana Dobreva, Mathias Heikenwalder, Soni Savai-Pullamsetti, Thomas Braun, Guillermo Barreto

**Affiliations:** 1 Lung Cancer Epigenetic, Max-Planck-Institute for Heart and Lung Research, Bad Nauheim, 61231 Germany; 2 Division Chronic Inflammation and Cancer (F180), German Cancer Research Center (DKFZ), Heidelberg, 69120 Germany; 3 Department of Lung Development and Remodeling, Max-Planck-Institute for Heart and Lung Research, Bad Nauheim, 61231 Germany; 4 Department of Cardiac Development, Max-Planck-Institute for Heart and Lung Research, Bad Nauheim, 61231 Germany; 5 Anatomy and Developmental Biology, CBTM, Heidelberg University, Mannheim, 68167 Germany; 6 European Center for Angioscience (ECAS), Medical Faculty Mannheim, Heidelberg University, Mannheim, 68167 Germany; 7 Pharmaceutical Technology and Biopharmaceutics, Department of Pharmacy, Ludwig-Maximilians-University of Munich, Munich, 81377 Germany; 8 Faculty of Medicine, Biochemistry Institute, Justus Liebig University, Giessen, 35392 Germany; 9 National Heart Research Institute, National Heart Centre Singapore, Singapore, 169609 Singapore; 10 Institute of Fundamental Medicine and Biology, Kazan (Volga Region) Federal University, Kazan, 420008 Russian Federation; 11 Tecnologico de Monterrey, Centro de Biotecnologia-FEMSA, Monterrey, 64849 NL Mexico; 12 Cardiovascular and Metabolic Disorders Program, Duke-National University of Singapore Medical School, Singapore, 169609 Singapore; 13 Chair for Lung Matrix Remodeling, Excellence Cluster Cardio Pulmonary System, Justus Liebig University, Giessen, 35392 Germany; 14 International Collaborative Center on Growth Factor Research, School of Pharmaceutical Sciences, Wenzhou Medical University and Institute of Life Sciences, Wenzhou University, Wenzhou, Zhejiang 325035 China; 15 Member of the Excellence Cluster Cardio Pulmonary System (ECCPS), The Universities of Giessen and Marburg Lung Center (UGMLC), Giessen, 35392 Germany; 16 German Center of Lung Research (Deutsches Zentrum für Lungenforschung, DZL), UGMLC, Giessen, 35392 Germany; 17 Department of General Pediatrics and Neonatology, University Children’s Hospital Giessen, Justus Liebig University, Giessen, 35392 Germany; 18 Institute for Pathology, Hanover Medical School, Hanover, 30625 Germany; 19 Biomedical Research in Endstage and Obstructive Lung Disease Hanover (BREATH) Research Network, Hanover, 30625 Germany; 20 Pulmonary and Critical Care Medicine, Department of Internal Medicine, Justus Liebig University, Giessen, 35392 Germany; 21 Agaplesion Lung Clinic Waldhof Elgershausen, Greifenstein, 35753 Germany; 22 Laboratoire Croissance, Réparation et Régénération Tissulaires (CRRET), CNRS ERL 9215, Université Paris Est Créteil, Université Paris Est, Créteil, F-94000 France

**Keywords:** Chromatin, Histone post-translational modifications, Non-coding RNAs, Transcription, Respiratory tract diseases

## Abstract

Idiopathic pulmonary fibrosis (IPF) is a chronic, progressive, and highly lethal lung disease with unknown etiology and poor prognosis. IPF patients die within 2 years after diagnosis mostly due to respiratory failure. Current treatments against IPF aim to ameliorate patient symptoms and to delay disease progression. Unfortunately, therapies targeting the causes of or reverting IPF have not yet been developed. Here we show that reduced levels of miRNA lethal 7d (*MIRLET7D*) in IPF compromise epigenetic gene silencing mediated by the ribonucleoprotein complex MiCEE. In addition, we find that hyperactive EP300 reduces nuclear HDAC activity and interferes with MiCEE function in IPF. Remarkably, EP300 inhibition reduces fibrotic hallmarks of in vitro (patient-derived primary fibroblast), in vivo (bleomycin mouse model), and ex vivo (precision-cut lung slices, PCLS) IPF models. Our work provides the molecular basis for therapies against IPF using EP300 inhibition.

## Abstract (web summary)

Idiopathic pulmonary fibrosis (IPF) is a lethal disease with insufficient treatment strategies. Here the authors show that reduction of the microRNA MIRLET7D and hyperactivation of EP300 contribute to impaired epigenetic silencing by the MiCEE complex in pulmonary fibroblasts of IPF patients, and demonstrate the benefit of inhibiting EP300 for the treatment of IPF.

## Introduction

Idiopathic pulmonary fibrosis (IPF) is the most common interstitial pulmonary disease showing a prevalence of 20 new cases per 100,000 persons per year^1,2^. Unfortunately, these numbers are rising due to increased lung injuries after exposure to air pollution, a consequence of industrialization^3–5^. A central event in IPF is the abnormal proliferation and migration of fibroblasts into the alveolar space after lung injury. Although under normal circumstances fibroblasts are important for wound healing and connective tissue production, their function in the fibrotic lung is out of control. It comes to formation of fibroblastic foci, which consist of highly proliferative fibroblasts, certain immune cells, and excessive extracellular matrix (ECM) protein deposition, such as fibronectin (FN1) and collagen (COL1A1). The consequences of these processes are disproportionate levels of scar tissue, alterations of the alveolar framework, changes in the lung epithelium structure, stiffening of the functional lung tissue, loss of the gas exchange function of the lung, and dramatically decreased oxygen saturation of the blood^2,6^.

Myofibroblasts are the key effector cell type that promotes remodeling of connective tissue after injury. Myofibroblasts are contractile-activated fibroblasts that express α-smooth muscle actin (ACTA2) to facilitate wound closure^7–10^. After wound healing, myofibroblasts can dedifferentiate into resting cells such as lipofibroblasts^9,11^. However, persistent activation of myofibroblasts causes excessive deposition of ECM proteins in the interstitial space leading to fibroblastic foci^5,12^. The precise control of gene expression is essential for both expansions of activated fibroblasts after injury and dedifferentiation into resting fibroblasts after wound healing. During dedifferentiation of activated fibroblast into resting fibroblast, the balance between transcription of lineage-specific genes and repression of pro-fibrotic genes allows resolution of fibroblastic foci. Transcriptional regulation of gene expression is closely linked to chromatin structure, which constitute the physiological template for all DNA-dependent biological processes in the cell nucleus. Chromatin structure is modulated by different processes including covalent modification of nucleotides on the DNA^13^, posttranslational histone modifications^14,15^, changes of nucleosome density by nucleosome remodeling or deposition/eviction^16^, changes in histone composition of nucleosomes^17^, activity of non-histone chromatin-associated proteins^18,19^, and gene regulation by noncoding RNAs (ncRNAs) including microRNAs (miRNAs, 21–25 nucleotides long) and long ncRNAs (>200 nucleotides long)^20^. Beyond its association to several human diseases, miRNAs are considered key diagnostic markers as well as dynamic regulators of phase separation as the organizing principle for tridimensional nuclear architecture^21^. Increasing evidence of mature miRNAs in the nucleus suggests a novel function in sub-nuclear compartments of mammalian cells^22–24^. In our previous work^25^ we characterize a multicomponent RNA–protein complex containing miRNA–ncRNA duplexes, the exosome-associated protein C1D, the nuclear-specific exosome subunit EXOSC10 (also known as RRP6), and the histone methyl transferase EZH2 (enhancer of zeste homolog 2). We demonstrate that this ribonucleoprotein complex, which we called MiCEE (*Mirlet7d*-C1D-EXOSC10-EZH2), mediates global genome organization and epigenetic silencing of bi-directionally expressed genes. In the present work we elucidate the clinical relevance of MiCEE using human lung tissue and primary fibroblasts, both isolated from control donors and IPF patients. The nuclear miRNA lethal 7d (*MIRLET7D*, also known as *let-7d*), is a functional component of MiCEE^25^. We demonstrate that reduced *MIRLET7D* levels in IPF compromised epigenetic silencing mediated by the MiCEE complex. In addition, we find that in control donors, deacetylation of histone 3 at lysine 27 (H3K27) mediated by histone deacetylase 1 and 2 (HDAC1 and HDAC2)^26^ anticipates methylation of the same residue (H3K27me3) during MiCEE-mediated heterochromatin formation. However, in IPF we detect hyperactive EP300 (E1A-binding protein p300, also known as P300)^27^, which inhibits nuclear HDAC1 and interferes with MiCEE function. Interestingly, we find reduced HDAC activity in the nucleus of IPF fibroblasts, which apparently is in contrast to previous reports^28–30^ that propose the use of HDAC inhibitors as potential treatment against pulmonary fibrosis. Remarkably, results after EP300 inhibition support our model and demonstrate reduced fibrotic hallmarks of in vitro (patient-derived primary fibroblast), in vivo (bleomycin mouse model), and ex vivo (precision-cut lung slices, PCLS) IPF models. Our study provides the molecular basis toward more efficient therapies against IPF using EP300 inhibition.

## Results

### Reduced *MIRLET7D* in IPF compromises MiCEE complex function

**Fig. 1:**
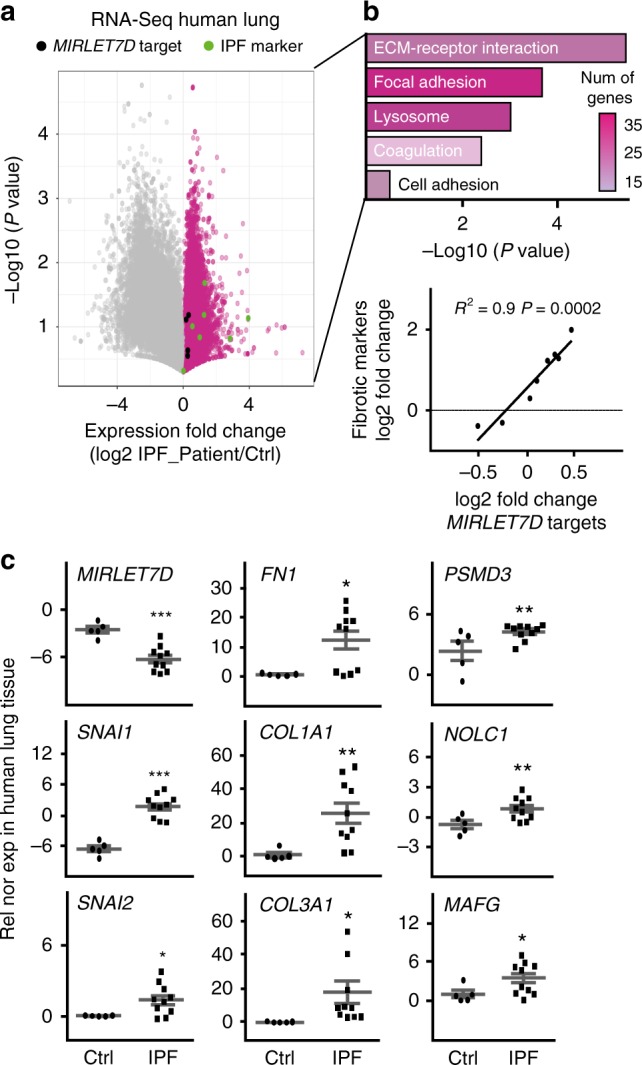
Nuclear *MIRLET7D* targets can be used as novel IPF markers. **a** RNA-sequencing in lung homogenates from Ctrl and IPF patients^31^. Volcano plot representing the significance (−log10 *P*-values after Welch’s *t*-test) vs. expression fold change (log2 expression ratios) between average of Ctrl and two IPF patients. Magenta dots show transcripts with positive log2 expression ratios. Black dots show *MIRLET7D* targets. Green dots show fibrotic markers. **b** Top: KEGG-based enrichment analysis of transcripts upregulated in both IPF patients (magenta dots in **a**) using DAVID bioinformatics tool and plotted by highest significance (−log10 of modified Fisher’s exact *P*-value). Color of the bar represents number of genes in each group based on the legend. ECM, extracellular matrix. Bottom: correlation analysis between *MIRLET7D* targets and fibrotic by linear regression of log2 FC value of a single *MIRLET7D* target paired with a single fibrotic marker from the two selected patients. All values were patient-matched and correlation clustering (data mining) from negative to positive values. **c** Mature *MIRLET7D*-specific TaqMan assay and qRT-PCR analysis of the indicated coding RNAs (cRNA) using total lung homogenates from Ctrl (*n* = 5 biologically independent samples) and IPF patients (*n* = 10 biologically independent samples). Data are shown as means ± SEM. Asterisks in all plots, *P*-values after unpaired *t*-test, one-tailed, ***P < 0.001; **P  < 0.01; *P < 0.05. Source data are provided as a Source Data file

Analysis of publically available RNA-sequencing (RNA-seq) data of lung tissue samples from IPF patients^31^ showed increased levels of fibrosis markers (Fig. 1a), including *FN1*, *COL1A1*, *ACTA2*, and snail family transcription repressors 1 (*SNAI1*, also known as Snail) and 2 (*SNAI2*, also known as Slug)^17^. KEGG (Kyoto Encyclopedia of Genes and Genomes) pathway analysis of the transcripts with increased expression in IPF patients (Fig. 1b, top) revealed statistically significant enrichment of pathways related to a fibrotic phenotype. Interestingly, we found a high positive correlation (Pearson’s *R*^2^ = 0.9; *P* = 0.0002; Fig. 1b, bottom) between the sample-to-sample expression (measured as a log2 FC value in the RNA-seq data) of fibrosis markers and genes that we recently identified as targets of *MIRLET7D* in the cell nucleus (*CHMP2B*, *MAFG*, *NOLC1*, and *PSMD3*; further referred as *MIRLET7D* targets)^25^. To confirm these results, we analyzed the expression of mature *MIRLET7D* and its targets by TaqMan assay and quantitative reverse transcriptase PCR (qRT-PCR) in lung tissue samples from control (Ctrl; *n* = 5) and IPF (*n* = 10) patients, respectively (Fig. 1c). We observed reduced *MIRLET7D* levels in IPF when compared with Ctrl human lung tissue, as previously reported^32^. Correlating with reduced *MIRLET7D* levels, we detected increased expression of *MIRLET7D* targets concomitant with high transcript levels of fibrosis markers. Our results confirmed that the recently identified *MIRLET7D* targets^25^ could be used as novel IPF markers.

**Fig. 2:**
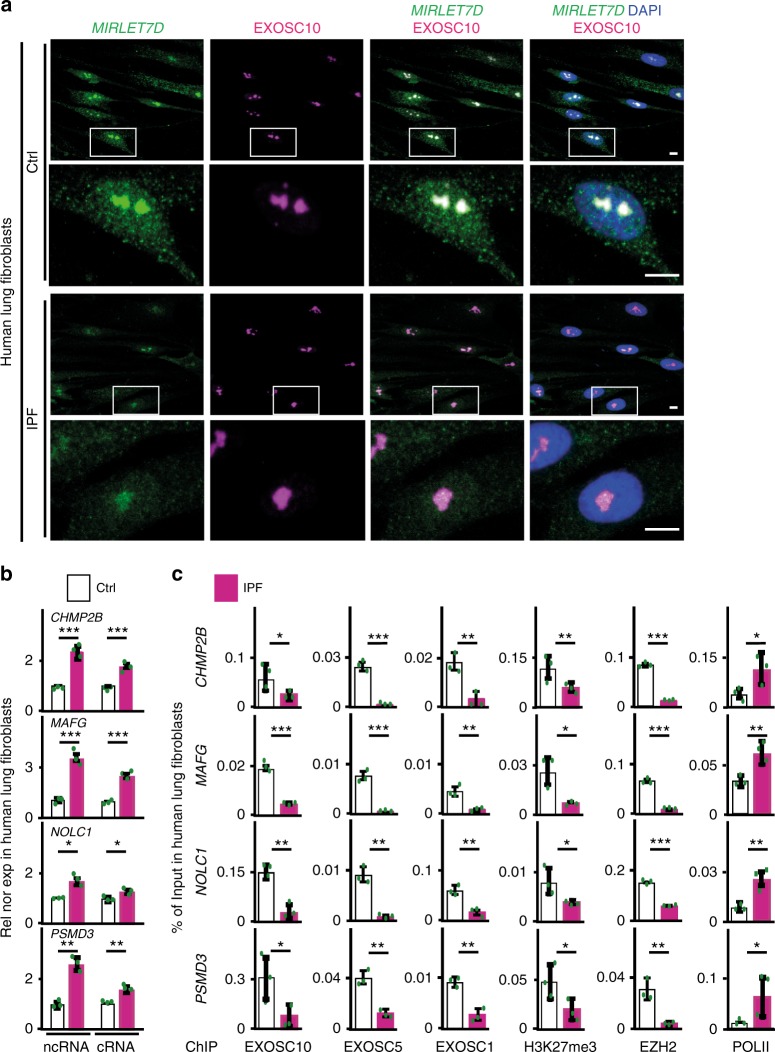
Reduced *MIRLET7D* in IPF compromises MiCEE complex function. **a** Confocal microscopy after *MIRLET7D*-FISH and EXOSC10 immunostaining shows nuclear colocalization of *MIRLET7D* and EXOSC10 in Ctrl primary human lung fibroblasts and reduced level of *MIRLET7D* in IPF primary human lung fibroblasts. DAPI, nucleus. Scale bars, 5 μm. Squares are respectively shown below at higher magnification. **b** qRT-PCR-based analysis of noncoding RNA (ncRNA) and cRNA of representative nuclear *MIRLET7D* targets^25^ in Ctrl or IPF primary lung fibroblasts. See Supplementary Figs. 1 and 2. **c** ChIP analysis of promoters from representative *MIRLET7D* targets using the indicated antibodies and chromatin isolated from Ctrl or IPF primary human lung fibroblasts. In all bar plots, data are shown as mean (SD) (*n* = 3 biologically independent experiments). Asterisks in all plots, *P*-values after unpaired *t*-test, two-tailed, ***P < 0.001; **P < 0.01; *P < 0.05; ns, not significant. Source data are provided as a Source Data file

In silico analysis of human *MIRLET7D* target loci (Supplementary Fig. 1a) revealed similar gene structures as in the mouse orthologs, which suggested transcriptional activity leading to the expression of ncRNA and corresponding mRNA from each locus^33,34^. To determine whether the ribonucleoprotein complex MiCEE^25^, in which *Mirlet7d* is functionally relevant, mediates epigenetic silencing in humans as it does in mice, we performed various experiments using primary fibroblasts isolated from lung tissue from Ctrl (*n* = 3) and IPF (*n* = 3) patients (Supplementary Fig. 1b). Combined *MIRLET7D*-specific fluorescence in situ hybridization (FISH) and EXOSC10-specific immunostaining (Fig. 2a) revealed co-localization of *MIRLET7D* and EXOSC10 in specific regions of the nucleus of human primary Ctrl fibroblasts. In addition, we detected reduced *MIRLET7D* levels in the nucleus and cytosol of IPF fibroblasts, which were further confirmed by TaqMan assay-based expression analysis after cellular fractionation (Supplementary Fig. 1c). RNA-seq in primary fibroblasts (Supplementary Fig. 2a–c) confirmed the RNA-seq results from human lung tissue (Fig. 1a), i.e., increased levels of *MIRLET7D* targets in IPF fibroblasts concomitant with fibrosis markers (Supplementary Fig. 2c, left). In addition, alternative mapping of our RNA-seq data to NONCODE database (Supplementary Fig. 2c, right) revealed increased expression of ncRNAs associated to *MIRLET7D* targets in IPF fibroblasts. Our RNA-seq in human primary fibroblasts was confirmed by expression analysis of representative *MIRLET7D* targets by qRT-PCR (Fig. 2b). Furthermore, promoter analysis of the same *MIRLET7D* targets by chromatin immunoprecipitation (ChIP; Fig. 2c) showed decreased levels of various subunits of the RNA exosome complex (EXOSC10, EXOSC5, and EXOSC1), the heterochromatin mark H3K27me3 (trimethylated Lys-27 of histone 3), and the enzyme mediating this histone modification (EZH2), whereas the levels of transcription initiating S5 phosphorylated RNA polymerase II (POLII) increased in IPF, compared with Ctrl fibroblasts.

**Fig. 3:**
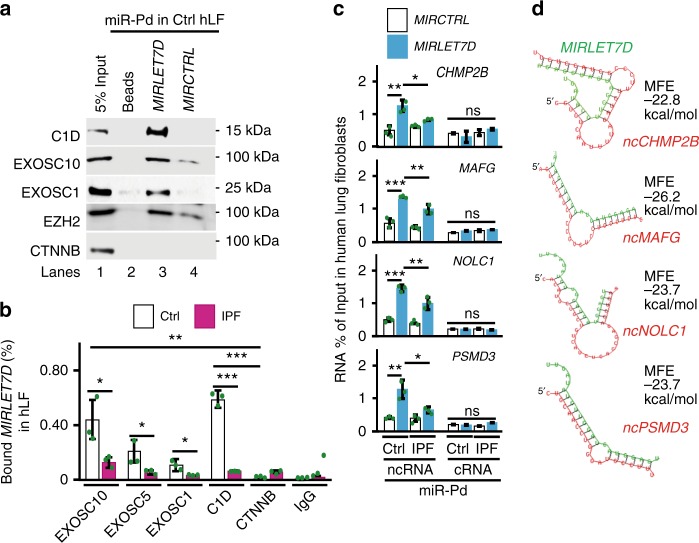
*MIRLET7D* binds the MiCEE complex and ncRNAs from *MIRLET7D* targets. **a** Western blotting (WB) of precipitated proteins after miRNA pulldown (miR-Pd) using nuclear fraction from Ctrl primary human lung fibroblasts (hLF) and biotinylated *MIRCTRL* or *MIRLET7D*. Input, 5% of starting material. Beads, control without probe. **b** Mature *MIRLET7D*-specific TaqMan assay after chromatin RNA immunoprecipitation (Ch-RIP) using the indicated antibodies and chromatin isolated from Ctrl or IPF hLF. CTNNB (beta-catenin) and IgG were used as negative controls. **c** qRT-PCR-based analysis of the indicated ncRNAs and cRNAs after miR-Pd from Ctrl or IPF hLF transfected with biotinylated *MIRCTRL* or *MIRLET7D*. **d** In silico analysis of ncRNAs from *MIRLET7D* target genes (magenta) identified *MIRLET7D* (green)-binding sites. MFE, minimum free energy. In all bar plots, data are shown as mean (SD) (*n* = 3 biologically independent experiments). Asterisks in all plots, *P*-values after unpaired *t*-test, two tailed, ***P < 0.001; **P < 0.01; *P < 0.05; ns, not significant. Source data are provided as a Source Data file

To further characterize the human MiCEE complex, we performed western blotting (WB) analysis of proteins after miRNA pulldown (miR-Pd) using the nuclear fraction of human Ctrl lung fibroblasts and biotinylated control miRNA (*MIRCTRL*) or *MIRLET7D* as bait (Fig. 3a). Several components of the MiCEE complex, including C1D, EXOSC10, EXOSC1, and EZH2, were specifically enriched after *MIRLET7D*-Pd. Notably, these results were confirmed by TaqMan-based miRNA enrichment analysis after chromatin RNA IP (Ch-RIP) using antibodies specific for different subunits of the MiCEE complex (Fig. 3b). All of the MiCEE subunits tested specifically bound mature *MIRLET7D* in Ctrl fibroblasts. In addition, *MIRLET7D* binding by MiCEE components decreased in IPF fibroblasts. To link these results to *MIRLET7D* targets, we analyzed them by miR-Pd using the nuclear fraction of Ctrl or IPF primary fibroblasts transfected with biotinylated *MIRCTRL* or *MIRLET7D* (Fig. 3c) and found that *MIRLET7D* exclusively pulled down ncRNAs and not mRNAs. These results confirmed the specific interaction of *MIRLET7D* with ncRNAs but not with mRNAs. Consistent with these results, in silico analysis of ncRNAs related to *MIRLET7D* targets identified *MIRLET7D*-binding sites with relatively favorable minimum free energy ( ≤ −20 kcal/mol; Fig. 3d). These results provide a plausible explanation for the increased ncRNA and mRNA levels observed in IPF fibroblasts. Although *MIRLET7D*-targeted and exosome-mediated degradation is required to reduce the levels of ncRNAs, transcriptional silencing after heterochromatin formation is required for the reduction of the corresponding mRNAs. Our results indicate that the MiCEE complex mediates epigenetic silencing in humans as it does in mice^25^ and its function is compromised in IPF due to reduced *MIRLET7D* levels.

**Fig. 4:**
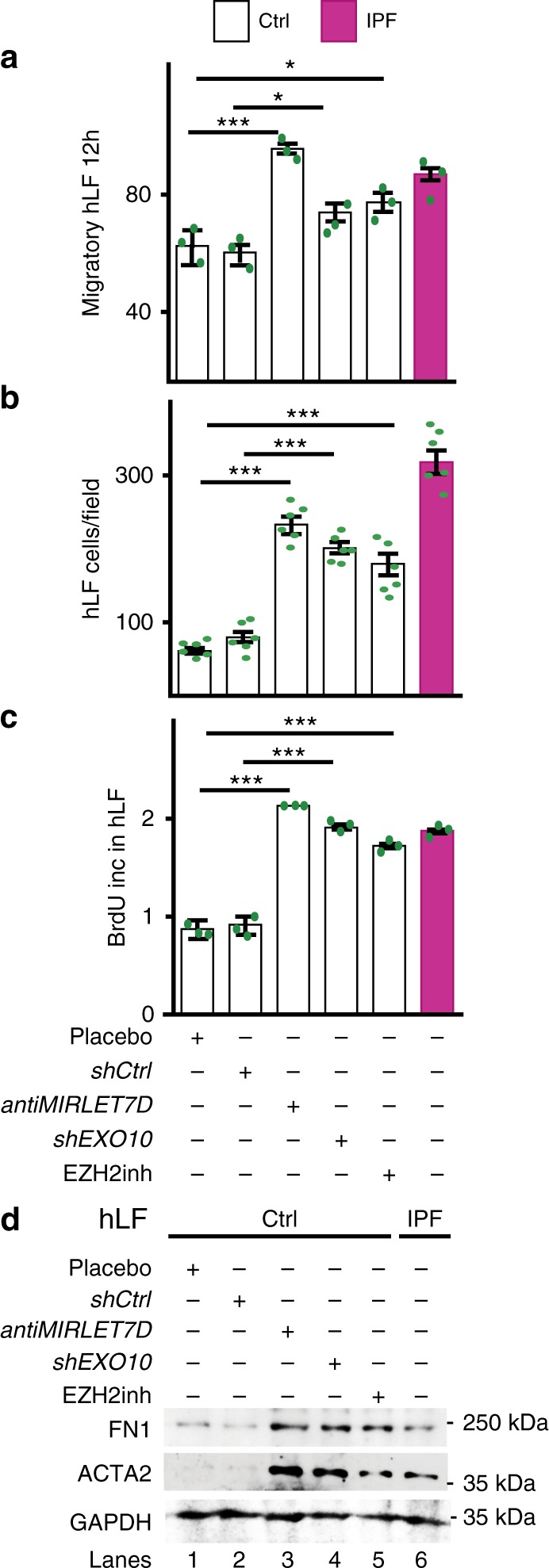
Loss-of-function of MiCEE components increased fibrosis hallmarks. **a**–**d** LOF of *MIRLET7D* by an antagomir (*antiMIRLET7D*), *EXOSC10* by a short hairpin RNA construct (*shEXO10*), and EZH2 by the small-molecule inhibitor UNC-1999 (EZH2inh) in Ctrl human primary lung fibroblasts (hLF) increased cell migration (**a**, **b**), cell proliferation (**c**), and protein levels of fibrotic markers (**d**) to similar levels as in IPF hLF. Scratch migration assays (**a**) of Ctrl or IPF hLF quantified as the migratory distance 12 h after scratch (see Supplementary Fig. 3a). Quantification of Transwell invasion assays followed by hematoxylin and eosin (H&E) stain (**b**) of Ctrl or IPF hLF (see Supplementary Fig. 3b). BrdU incorporation assays (**c**) using Ctrl or IPF hLF. WB of whole-cell protein extracts from Ctrl or IPF hLF using the indicated antibodies (**d**). In all bar plots, data are shown as mean (SD) (*n* = 3 biologically independent experiments for **a** and **c**; *n* = 6 biologically independent experiments for **b**). Asterisks in all plots, *P*-values after unpaired *t*-test, two tailed, ***P < 0.001; **P < 0.01; *P < 0.05; ns, not significant. Source data are provided as a Source Data file

Our results indicate that the MiCEE complex is required for proper function of human lung fibroblasts. To test this hypothesis, we monitored the effects of loss-of-function (LOF) of different MiCEE components on fibrosis hallmarks (Fig. 4). LOF of *MIRLET7D* (by antagomiR probes)^32^, EXOSC10 (by short hairpin RNA construct, shRNA)^25^, and EZH2 (by small-molecule inhibitor, UNC-1999)^35^ in Ctrl human primary lung fibroblasts increased cell migration (Fig. 4a, b and Supplementary Fig. 3a, b), cell proliferation (Fig. 4c), and levels of fibrotic markers (Fig. 4d) to similar levels as in IPF fibroblasts, thereby demonstrating the requirement of the MiCEE complex for appropriate lung fibroblast functions.

### Nuclear HDAC activity is reduced in IPF

**Fig. 5:**
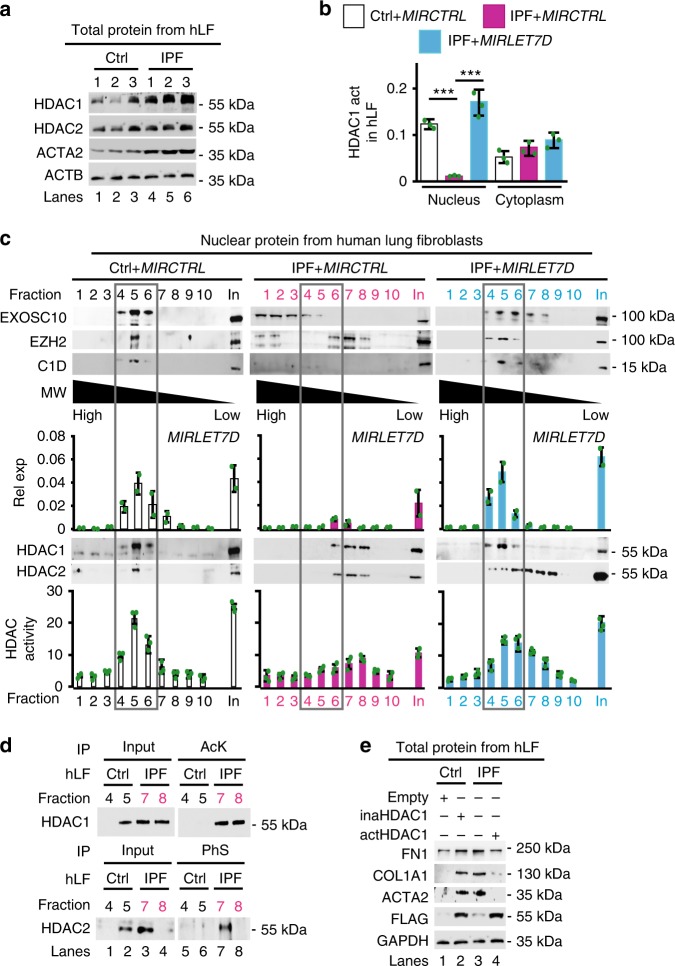
Nuclear histone deacetylase activity is reduced in IPF. **a** Western blot (WB) using the indicated antibodies and whole-cell protein extracts from Ctrl and IPF primary human lung fibroblasts (hLF) derived from three different donors, each. **b** HDAC1-specific activity assay using nuclear or cytosolic protein extracts from Ctrl or IPF hLF that were transfected with control miRNA (*MIRCTRL*) or *MIRLET7D* probes. **c** WB using antibodies specific for MiCEE components (top) or HDAC1, or 2 (middle), *MIRLET7D*-specific TaqMan assay (middle), and fluorometric HDAC activity assay (bottom) of fractions after sucrose gradient ultracentrifugation (SGU) of nuclear extracts from Ctrl and IPF hLF transfected as in **b**. Squares show fractions where MiCEE is expected. In, Input (5% of material prior SGU); MW, molecular weight; Rel exp, relative expression. **d** WB using HDAC1 (top) or HDAC2 (bottom)-specific antibodies after immunoprecipitation using either pan-acetyl-lysine (AcK) or phospho-serine (PhS)-specific antibodies to precipitate endogenous inactive HDAC1 and HDAC2 from selected SGU fractions analyzed in **c**. Input, 5% of IP starting material. **e** WB using the indicated antibodies and whole-cell protein extracts from Ctrl and IPF hLF that were transfected with an empty plasmid or expression constructs containing inactive (ina) or active (act) HDAC1 as indicated. In all bar plots, data are shown as mean (SD) (*n* = 3 biologically independent experiments for **a** and **c**; *n* = 2 biologically independent experiments for **b**). Asterisks in all plots, *P*-values after unpaired *t*-test, two tailed, ***P < 0.001; **P < 0.01; *P < 0.05; ns, not significant. Source data are provided as a Source Data file. See Supplementary Fig. 4

Similar to MiCEE, HDAC1 and HDAC2 are commonly linked to heterochromatin formation^26^. In addition, increased levels of HDACs have been shown in human lung tissue from IPF patients^28–30^. Thus, we decided to analyze the levels and the enzymatic activity of HDACs in protein extracts from patient-derived Ctrl and IPF lung fibroblasts. WB analysis of total protein extracts showed increased levels of HDAC1 and 2 in IPF as compared with Ctrl fibroblasts (Fig. 5a), correlating with previous reports^28–30^. However, two types of HDAC activity assays (Fig. 5b and Supplementary Fig. 4a) using nuclear and cytosolic extracts from Ctrl or IPF fibroblasts detected reduced HDAC activity in the nucleus of IPF fibroblasts when compared with Ctrl fibroblasts. Interestingly, *MIRLET7D* gain-of-function (GOF) in IPF fibroblasts reconstituted nuclear HDAC activity. On the other hand, cytosolic HDAC activity was higher in IPF fibroblasts compared with Ctrl fibroblasts. These results together indicated that HDAC1 and 2, although present in IPF nuclear extracts, might be inactive. To prove this hypothesis, nuclear extracts from Ctrl and IPF primary fibroblasts were fractionated by sucrose gradient ultracentrifugation (SGU) (Fig. 5c). Analysis of the obtained fractions by WB and TaqMan assay (top and middle) revealed that EXOSC10, EZH2, C1D, and *MIRLET7D* co-sedimented in fraction 5 of Ctrl nuclear extracts, supporting proper formation of the MiCEE complex. Interestingly, in IPF nuclear extracts the levels of *MIRLET7D* and C1D were reduced, and EXOSC10 and EZH2 sedimented in different fractions, suggesting a disruption of this complex. In addition, we analyzed SGU fractions by WB using HDAC1- and HDAC2-specific antibodies (bottom) and detected both deacetylases in the same fraction as MiCEE in Ctrl nuclear extracts. In contrast, HDAC1 and HDAC2 were detected in IPF nuclear extracts mainly in fractions 7 to 9. Further analysis of SGU fractions by HDAC activity assays showed enrichment of deacetylase activity in fraction 5 of Ctrl nuclear extracts, whereas reduced HDAC activity was observed in IPF nuclear extracts. Strikingly, *MIRLET7D* transfection into IPF fibroblasts (Fig. 5c, right) was sufficient to reconstitute the MiCEE complex and the HDAC activity almost to similar levels as in Ctrl fibroblasts. The reconstituting effect of *MIRLET7D*-GOF on the MiCEE complex was confirmed by Co-IP of nuclear protein extracts from IPF lung fibroblasts using C1D-specific antibodies (Supplementary Fig. 4e). To further confirm the reduction of nuclear HDAC activity in IPF, we analyzed fractions containing HDAC1 and HDAC2 by IP assays of endogenous proteins using antibodies specific for pan-acetylated lysine (AcK) or phosphorylated serine (PhS) and subsequent WB analysis of precipitated proteins using HDAC1- or HDAC2-specific antibodies (Fig. 5d). It has been reported that HDAC1 acts upstream of HDAC2 and inactivation of HDAC1 leads to inactivation of HDAC2^36^. Acetylation of specific lysine residues (K218, K220, K432, K438, K439, and K441) inactivates HDAC1^37^, whereas phosphorylation of specific serine residues (S394, S422, and S424) subsequently inactivates HDAC2^38^. Correlating with our HDAC activity assays (Fig. 5b, c), we detected inactive (AcK) HDAC1 and (PhS) HDAC2 in fractions 8 and 9 of IPF nuclear extracts, but not in fractions 5 and 6 of Ctrl nuclear extracts. The presence of inactive HDAC1 in the nucleus of IPF fibroblasts was also confirmed by proximity ligation assays (PLAs, Supplementary Fig. 4b) using AcK- and HDAC1-specific antibodies. Our results together confirmed that HDAC1 and HDAC2 are inactive in the nucleus of IPF fibroblasts. To further determine the role of HDAC1 activity, we transfected Ctrl primary fibroblasts with a constitutively inactive HDAC1 mutant (inaHDAC1), in which specific lysine residues were mutated to glutamine (K218Q, K220Q, K432Q, K438Q, K439Q, and K441Q), mimicking AcK^37^. WB analysis of protein extracts from transfected Ctrl fibroblasts (Fig. 5e, lanes 1–2) showed increased levels of FN1, COL1A1, and ACTA1 after overexpression of inaHDAC1. The levels of these fibrosis markers in transfected Ctrl fibroblasts were similar to non-transfected IPF fibroblasts (compare lanes 2 and 3). Interestingly, transfection of IPF fibroblasts with a hyperactive HDAC1 mutant (actHDAC1), in which specific lysine residues were mutated to arginine (K218R, K220R, K432R, K438R, K439R, and K441R) to avoid AcK^37^, reduced the levels of the fibrosis markers (lanes 3–4) to levels similar to non-transfected Ctrl fibroblasts (compare lanes 4 and 1). To link the results obtained using protein extracts from primary fibroblasts with the *MIRLET7D* target genes, we analyzed these genes by ChIP, ChIP–reChIP (ChIP and sequential ChIP) (Supplementary Fig. 4c, d and Supplementary Discussion). These results demonstrated accumulation of inactive HDAC1 and HDAC2 at the promoters of *MIRLET7D* targets in IPF fibroblasts (Supplementary Fig. 4c, d). All these results together indicate that reduced nuclear HDAC activity in IPF interferes with proper MiCEE function and is crucial for fibrosis.

It has been previously suggested that removal of the acetyl group from H3K27 has to take place before the same amino acid residue can be methylated^39^. To confirm this sequential order of events in our experimental system, we analyzed the promoters of *MIRLET7D* targets by ChIP using antibodies specific for acetylated H3K27 (H3K27ac) or trimethylated H3K27 (H3K27me3) and chromatin isolated from IPF fibroblasts after *MIRCTRL* or *MIRLET7D* transfection alone or in combination with shRNAs specific for *HDAC1* (*shHDAC1*; Supplementary Fig. 5a) or *EZH2* (*shEZH2*; Supplementary Fig. 5b). Consistent with our previous results^25^, *MIRLET7D*-GOF reduced the levels of the euchromatin mark H3K27ac, whereas the levels of the heterochromatin mark H3K27me3 increased. Interestingly, although *shHDAC1* counteracted the effect of *MIRLET7D* on both chromatin marks, *shEZH2* only counteracted the effect of *MIRLET7D* on H3K27me3. Our results support that HDAC1-mediated deacetylation of H3K27 is required and anticipates EZH2-mediated methylation of the same amino acid residue, thereby confirming previously suggested models^39^. Furthermore, expression analysis of *MIRLET7D* targets by qRT-PCR (Supplementary Fig. 5c) correlated with the chromatin changes detected by ChIP analysis (Supplementary Fig. 5a, b).

### Active EP300 inactivates HDAC1 in IPF

**Fig. 6:**
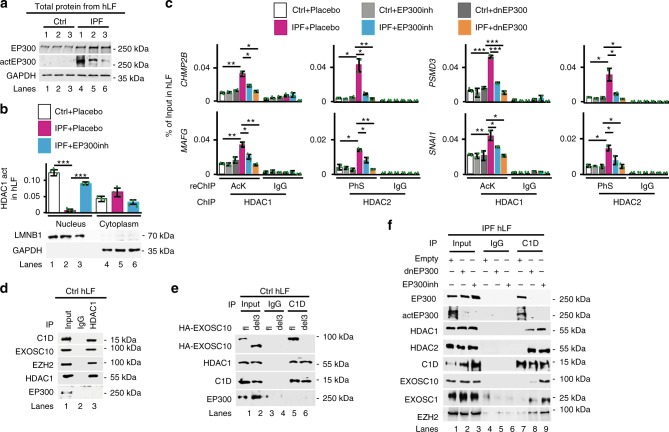
Active EP300 inactivates HDAC1 in IPF. **a** WB using the indicated antibodies and whole-cell protein extracts from Ctrl and IPF lung fibroblasts (hLF) derived from three different donors each. ActEP300, active EP300. See Supplementary Fig. 6a–c. **b** Top: HDAC1-specific activity assay using nuclear or cytosolic protein extracts from Ctrl or IPF hLF treated with placebo (vehicle solution) or 10 µM EP300 inhibitor (EP300ihn, CBP30). Bottom: WB monitoring the quality of the fractions used in the HDAC1 activity assay. LMNB1, nuclear marker. GAPDH, cytosolic marker. See Supplementary Figs. 6d and 7a. **c** Sequential ChIP (ChIP–reChIP) analysis of representative *MIRLET7D* target promoters using HDAC1- or HDAC2-specific antibodies, followed by AcK- or PhS-specific antibodies, respectively, and chromatin isolated from Ctrl or IPF hLF after treatment with EP300inh or transfection of a dominant-negative EP300 (dnEP300) construct. IgG, immunoglobulin G (negative control). See Supplementary Fig. 7. **d** WB using the indicated antibodies after Co-IP assay using immunoglobulin G (IgG; negative control) or HDAC1-specific antibodies to immunoprecipitate endogenous HDAC1 from nuclear protein extracts of Ctrl hLF. Input, 5% of IP starting material. **e** WB after Co-IP assay as in **d** using C1D-specific antibodies to immunoprecipitate endogenous C1D from nuclear protein extracts of Ctrl hLF that were transfected with full-length (fl) or deletion mutant 3 (del3, lacking C1D interaction domain on amino acids 1–285) HA-tagged EXOSC10 constructs^25^. **f** WB after Co-IP assay as in **d** using C1D-specific antibodies to immunoprecipitate endogenous C1D from nuclear protein extracts of IPF hLF that were either transfected with empty plasmid or with dnEP300 expression construct or treated with EP300inh as indicated. In all plots, data are shown as mean (SD) (*n* = 3 biologically independent experiments in **b**; *n* = 2 biologically independent experiments in **c**); asterisks, *P*-values after unpaired *t*-test, two tailed, ***P < 0.001; **P < 0.01; *P < 0.05. Source data are provided as a Source Data file

It is known that EP300 acetylates HDAC1, thereby inactivating its HDAC activity^37^. Interestingly, RNA-binding modulates EP300 acetyltransferase activity^40^. Thus, we monitored the levels of total EP300 by WB in protein extracts from Ctrl and IPF fibroblasts (Fig. 6a and Supplementary Fig. 6a) and detected a slight increase of EP300 in IPF compared with Ctrl fibroblasts. Strikingly, detection of active EP300 (actEP300) using antibodies specific for EP300 phosphorylated at serine 1834^41^ showed remarkably increased actEP300 levels in IPF fibroblasts compared with Ctrl. Our WB results were confirmed by confocal microscopy of primary fibroblasts after immunostaining using antibodies specific for actEP300 (Supplementary Fig. 6b). Further, we analyzed the promoters of *MIRLET7D* targets by ChIP using EP300- or actEP300-specific antibodies (Supplementary Fig. 6c, left). In *MIRCTRL*-transfected IPF fibroblasts, we detected increased levels of both total EP300 and actEP300 in all promoters analyzed when compared with *MIRCTRL*-transfected Ctrl fibroblasts. Interestingly, *MIRLET7D*-GOF in IPF fibroblasts did not significantly change the levels of total EP300, but reduced the levels of actEP300, suggesting inactivation of EP300 rather than its release from promoters. This observation was confirmed by WB of protein extracts from IPF fibroblasts after *MIRLET7D*-GOF (Supplementary Fig. 6a, right), as well as by detection of inactive EP300 (inaEP300) by ChIP of *MIRLET7D* targets using antibodies specific for phosphorylated EP300 at serine 89^42^ (Supplementary Fig. 6c, right). To demonstrate the causal involvement of actEP300 during the inactivation of HDAC1 in IPF fibroblasts, we analyzed HDAC activity in nuclear protein extracts from Ctrl and IPF fibroblasts (Fig. 6b and Supplementary Fig. 6d). We detected reduced HDAC activity in the nucleus of IPF fibroblasts when compared with Ctrl fibroblasts, confirming our previous results (Fig. 5b, c). Interestingly, treatment of IPF fibroblasts with the EP300 inhibitor CBP30^43^ (EP300inh; Supplementary Fig. 7a) reconstituted the HDAC activity to levels similar to Ctrl fibroblasts, supporting the causal involvement of actEP300 during the inactivation of HDAC1 in IPF. Further analysis of *MIRLET7D* targets by ChIP (Supplementary Fig. 7c), ChIP–reChIP (Fig. 6c), and qRT-PCR (Supplementary Fig. 7d) in Ctrl and IPF fibroblasts, in which two methods of EP300-LOF were used, EP300inh treatment or transfection of dominant-negative EP300 (dnEP300; Supplementary Fig. 7a, b)^44^, clearly demonstrated the causal involvement of actP300 during HDAC inactivation in the nucleus of IPF fibroblasts. These results together demonstrate a key role of active EP300 mediating inactivation of nuclear HDAC in IPF.

Analysis of SGU fractions (Fig. 5c) strongly suggested an interaction between HDAC1 and the MiCEE complex. This interpretation was confirmed by Co-IP assay using nuclear protein extracts from Ctrl primary fibroblasts (Fig. 6d). Endogenous HDAC1 co-immunoprecipitated C1D, EXOSC10, and EZH2, demonstrating the interaction with functional components of the MiCEE complex. To further investigate these interactions, we analyzed by Co-IP assay nuclear protein extracts from Ctrl fibroblasts that were transfected either with EXOSC10 full-length (fl) or a deletion mutant lacking the amino acids 1–285 (del3), thereby losing the ability to interact with C1D^25^ (Fig. 6e). Endogenous C1D co-immunoprecipitated HDAC1 in the presence of both, EXOSC10 fl and del3, supporting a direct C1D-HDAC1 interaction that does not require the previously described C1D-EXOSC10 interaction^25^. Interestingly, neither HDAC1 (Fig. 6d) nor C1D (Fig. 6e) immunoprecipitated EP300 in Ctrl fibroblasts. However, Co-IP assay using nuclear protein extracts from IPF fibroblasts (Fig. 6f) revealed that C1D interacts with active EP300, whereas C1D-HDAC1 interaction was abolished (lane 7). Strikingly, two methods of EP300-LOF in IPF fibroblasts (EP300inh treatment and dnEP300 transfection; lanes 8 and 9) disrupted the C1D-EP300 interaction and reconstituted the C1D-HDAC1 interaction. Furthermore, the interaction of C1D with the other components of the MiCEE complex (EXOSC10, EXOSC1, and EZH2) was reconstituted after both methods EP300-LOF, supporting the appropriate formation of the MiCEE complex.

### EP300inh counteracts fibrosis hallmarks in IPF models

**Fig. 7:**
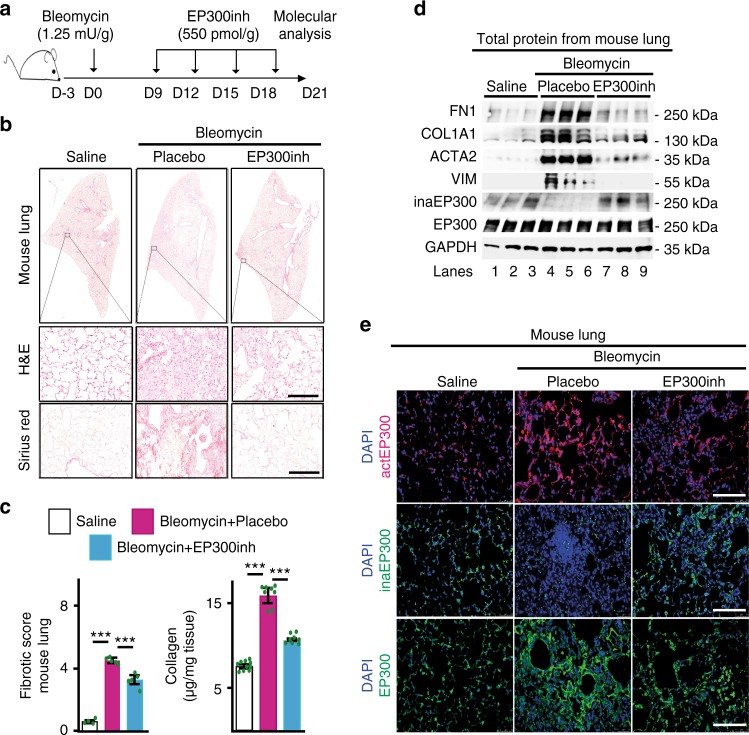
EP300 inhibition counteracts fibrosis hallmarks in bleomycin mouse model. **a** Scheme shows experimental setup. Three group of mice (control group *n* = 4, experimental groups *n* = 5, in each group biologically independent animals) were orotracheally instilled with saline or bleomycin (1.25 mU per g body weight) at day 0 and subsequently orotracheally instilled with placebo (vehicle solution) or EP300 inhibitor (EP300inh, CBP30, 550 pmol per g body weight) on days 9, 12, 15, and 18. Mice were collected for analysis at day 21. **b** Top: representative histological analysis using hematoxylin and eosin (H&E) stain on left lung sections from mice treated as in **a**. Squares are shown below at higher magnification. Bottom, representative light microscopy after Sirius red staining monitoring collagen deposition on left lung sections from mice treated as in **a**. Scale bar = 100 μm. See Supplementary Fig. 8. **c** Left: fibrotic (Ashcroft) score quantification from representative Sirius red staining on left lung sections from mice treated as in **a**. Right, collagen content quantification by Sircol assay using lung homogenates from mice treated as in **a**. Data are shown as mean (SD) (*n* = 10, 8, or 7 biologically independent samples); asterisks, *P*-values after unpaired *t*-test, two tailed, ***P < 0.001. Source data are provided as a Source Data file. **d** WB using the indicated antibodies and lung homogenates from three representative mice from each experimental group described in **a**. **e** Confocal microscopy after immunostaining using EP300, actP300, or inaEP300-specific antibodies in lung sections from representative mice from each experimental group described in **a**. ActEP300, active EP300; inaEP300, inactive EP300. DAPI, nuclear staining. Scale bar, 500 µm

**Fig. 8:**
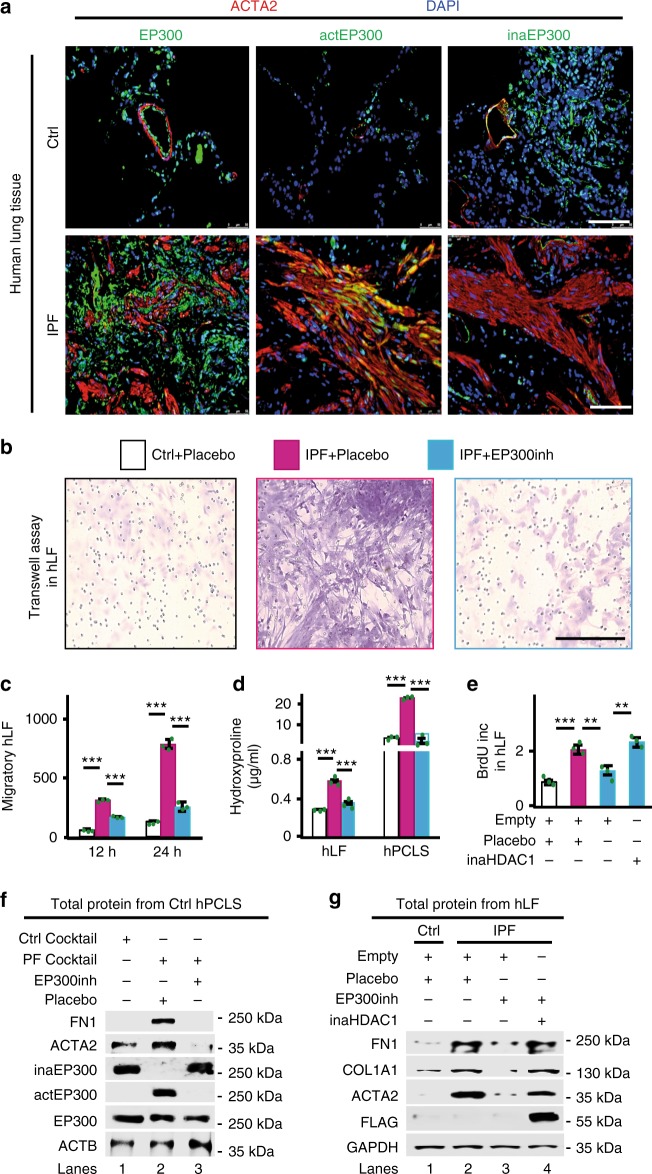
EP300inh counteracts fibrosis hallmarks in IPF models. **a** Confocal microscopy after ACTA2, EP300, actEP300, and inaEP300 immunostaining in representative lung sections from Ctrl and IPF patients shows actEP300 increase in IPF. ActEP300, active EP300. DAPI, nucleus. Scale bars, 500 μm. **b** Transwell invasion assay followed by hematoxylin and eosin (H&E) stain in human Ctrl or IPF lung fibroblasts (hLF) treated with placebo or EP300inh as indicated. Scale bar, 200 μm. **c** Scratch migration assay of Ctrl and IPF hLF treated as in **b** was quantified as the migratory distance 12 and 24 h after scratch. **d** Hydroxyproline assay for collagen content in hLF and human precision-cut lung slices (hPCLS) treated as in **b**. **e** Proliferation assay by BrdU incorporation using Ctrl or IPF hLF treated with placebo or EP300inh and/or transfected with empty plasmid or inactive HDAC1 expression construct as indicated. **f** WB using the indicated antibodies and whole-cell protein extracts from hPCLS that were treated with Ctrl or pro-fibrotic (PF) cocktail and placebo or EP300inh as indicated. **g** WB using the indicated antibodies and whole-cell protein extracts from Ctrl or IPF hLF treated as in **e**. In all bar plots, data are shown as mean (SD) (*n* = 3 biologically independent experiments); asterisks, *P*-values after unpaired *t*-test, two tailed, ***P < 0.001. Source data are provided as a Source Data file

**Fig. 9:**
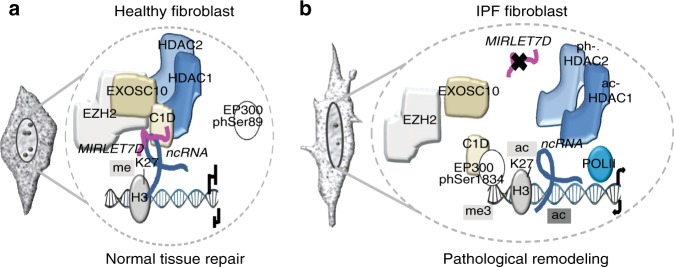
Model. **a** In healthy lung fibroblasts, C1D-bound *MIRLET7D*-ncRNA duplexes attract active HDAC1/2 and the MiCEE complex to specific loci. Active HDAC1/2 deacetylate H3K27, whereas EZH2 mediates trimethylation of H3K27, thereby leading to heterochromatin formation and transcriptional silencing. **b** In IPF lung fibroblasts, reduced *MIRLET7D* and active EP300, which inactivates HDAC1/2 and interacts with C1D, disrupt the MiCEE complex interfering with heterochromatin formation and transcriptional silencing at specific loci

Our results in primary fibroblasts and lung tissue support a key role of active EP300 in IPF. We therefore decided to determine the effect of EP300 inhibition on hallmarks of fibrosis in vivo (bleomycin mouse model, Fig. 7a–e and Supplementary Fig. 8a), ex vivo (PCLS, Supplementary Fig. 8b–d, Fig. 8d and 8f), and in vitro (human primary fibroblasts; Fig. 8b–e and 8g). In all these models of IPF, we detected upon EP300 inhibition reduced extracellular matrix protein deposition (Fig. 7b, c; Supplementary Fig. 8c, Fig. 8a and 8d), reduced levels of fibrotic markers, such as FN1, COL1A1, and ACTA2 (Fig. 7d; Supplementary Fig. 8d and Fig. 8f), and reduced cell migration and proliferation (Fig. 8b, 8c and 8e). All these results demonstrate the anti-fibrotic effects of EP300inh. Strikingly, transfection of constitutively inaHDAC1 mutant counteracted EP300inh (Fig. 8e and 8g), thereby supporting the causal involvement of active HDAC1 during the anti-fibrotic effects elicited by EP300 inhibition. Our results support a model in which HDAC1 interacts with the MiCEE complex via C1D in the nucleus of human Ctrl fibroblasts (Fig. 9a). In addition, we found that HDAC-mediated histone deacetylation is required and anticipates MiCEE-mediated heterochromatin formation. However, in the nucleus of human IPF fibroblasts (Fig. 9b), we detected both increase of active EP300, which inactivates HDAC1 and disrupts HDAC1-C1D interaction, and reduced levels of *MIRLET7D*, which compromises MiCEE-mediated epigenetic gene silencing. Further dissecting the part of our model, in which reduced HDAC1 activity was detected in the nucleus of IPF fibroblasts (Fig. 5b, c), we also found that inaHDAC1 is sufficient to increase the levels of fibrotic markers in Ctrl fibroblasts (Fig. 5e). These results support a key role of HDAC inactivation during IPF. Interestingly, EP300-LOF experiments (Fig. 6b, c and Supplementary Fig. 7a–d) demonstrated the requirement of actP300 for HDAC inactivation in IPF fibroblasts. Moreover, overexpression of inaHDAC1 counteracted the anti-fibrotic effects of EP300inh (Fig. 8e and 8g). Our results clearly support the causal involvement of active EP300 in mediating the inactivation of nuclear HDAC1 during IPF.

## Discussion

Previous reports have shown increased levels of HDACs in human lung tissue from IPF patients^28–30^, as we have detected in total protein extracts from IPF fibroblasts (Fig. 5a). However, these works also reported an increased HDAC activity in IPF. These apparent discrepancies could be explained by differences in the experimental systems. We have determined the enzymatic activity of HDACs in the nucleus, as well as HDAC1-specific enzymatic activity in the nucleus and cytosol of human primary fibroblasts from Ctrl and IPF patients, whereas the other reports neither discriminated between the cytosolic and nuclear fractions of cells used, nor directly determined the enzymatic activity stage of the HDACs. We detected reduced HDAC activity in the nucleus of IPF fibroblasts, whereas HDAC activity was increased in the cytosol of IPF fibroblasts (Fig. 5b, c and Supplementary Fig. 4a). Our work has implications on the proposed use of HDAC inhibitors as potential treatment against pulmonary fibrosis^28–30^. Whereas inactivation of cytosolic HDAC might be favorable for IPF patients, inactivation of nuclear HDAC might be counterproductive. Consistently with our hypothesis, it has been reported that activation of the NAD + -dependent HDAC SIRT1 (Silent information regulator type 1) attenuated lung fibrosis^45,46^. Thus, a therapy against IPF based on EP300 inhibition might be more specific by targeting and reconstituting nuclear HDAC1 activity. Supporting this line of ideas, EP300 inhibition has been shown to ameliorate fibrosis in other organs^47^. Furthermore, inhibition of the EP300-related protein CBP (CREB-binding protein) reversed pulmonary fibrosis^48^. Chromatin is the physiological template for all DNA-dependent processes including transcription, replication, recombination, and repair^49^. However, the molecular mechanisms underlying the changes in chromatin structure mediating transcriptional regulation after EP300 inhibition in fibrosis was sparsely investigated prior to our study. The EP300inh used here is a potent small-molecule inhibitor of the bromodomain-containing transcription factor EP300^50^ (Supplementary Fig. 9). The results presented in this manuscript using EP300inh in vitro (Fig. 8b–e, 8g, Supplementary Fig. 7), in vivo (Fig. 7a–e), and ex vivo (Fig. 8d, 8f, and Supplementary Fig. 8) strongly suggest that inhibition of the bromodomain is crucial for the role of EP300 during IPF. In addition, the results obtained after overexpression of dnEP300, which is an EP300 mutant lacking acetyltransferase activity due to an inactivating D1399Y point mutation^44^, complemented and confirmed the results obtained by EP300inh treatment. Moreover, we observed increased levels of acetylated H3 in IPF fibroblasts (Supplementary Fig. 5d), suggesting that HDAC1 inactivation by acetylation might be just one of the effects caused by active EP300 during IPF progression. However, subsequent mechanistic analysis elucidating the function of EP300 during IPF initiation and progression will be the scope of future work.

IPF is a fatal interstitial lung disease for which no cure exists. Although two drugs have been approved for the treatment of IPF in several countries^51,52^, the prognosis of IPF patients remains poor. Thus, the search for drugs against IPF continues and several compounds have been approved for Phase II development for treatment against IPF^53^. Remarkably, we demonstrated that EP300 inhibition reduced fibrotic hallmarks in vitro using primary fibroblasts from Ctrl and IPF patients, in vivo using the bleomycin mouse model, and ex vivo using PCLS. Our study lays the groundwork towards more efficient therapies targeting the causes of IPF using EP300 inhibition.

## Methods

### Study design

This study was performed according to the principles set out in the WMA Declaration of Helsinki; the underlying protocols were approved by the ethics committee of Medicine Faculty of the Justus Liebig University in Giessen, Germany (AZ.111/08-eurIPFreg) and the Hannover Medical School (number 2701–2015). In this line, all patient and control materials were obtained through the European IPF Registry (www.pulmonary-fibrosis.net), the UGMLC Giessen Biobank (member of the DZL Platform Biobanking), and the Biobank from the Institute for Pathology of the Hannover Medical School as part of the BREATH Research Network. All participants provided informed written consent in their respective center of enrollment. We used anonymized patient material. Patient characteristics are summarized in the Supplementary Fig. 1b.

Animal experiments were performed in accordance with German animal protection laws and were approved by the local governmental animal protection committee (Regierungspräsidium Darmstadt, Germany, Veterinärdezernat, approval number B2/1165). Eight‐ to 10‐week‐old male mice were orotracheally instilled with 2.5 U/kg body weight of bleomycin (Medac, Germany) that was dissolved in saline. Control groups were instilled with saline. Bleomycin-instilled mice were orotracheally instilled with Placebo or EP300inh (11 nmol/20 g body weight) on d9, d12, d15 and d18.

### Quantification and statistical analysis

Depending on the data, different tests were performed to determine the statistical significance of the results. The values of the statistical tests used in the different experiments can be found in the Statistical Summary (Source Data file). Further details of statistical analysis in different experiments are included in the figures and figure legends. Briefly, two biological replicates of RNA-seq were analyzed by deep sequencing. For the rest of the experiments presented here, samples were analyzed at least in triplicates and experiments were performed three times. Statistical analysis was performed using Excel Solver and Graph Prism (v.5). All data are represented as mean ± SD. *T*-test was used to determine the levels of difference between the groups and *P*-values for significance. *P*-values after *T*-test, *P *≤* 0.05; **P < 0.01, and ***P *<* 0.001.

### Cell culture, transfection, and treatment

Primary fibroblast from Ctrl and IPF patients were cultured in complete MCDB131 medium (8% fetal calf serum (FCS), 1% l‐glutamine, penicillin 100 U/ml, streptomycin 0.1 mg/ml, epidermal growth factor 0.5 ng/ml, basic fibroblast growth factor 2 ng/ml, and insulin 5 μg/ml)) at 37 °C in 5% CO_2_. Because of the concern that the phenotype of the cells is altered at higher passage, cells between passages 4 and 6 were utilized in the experiments described here, except cells for screening were used at earlier passages. During subculturing, cells were washed with 1× phosphate-buffered saline (PBS), trypsinized with 0.25% (w/v) trypsin, and subcultivated at the ratio of 1:5 to 1:10. Cells were transfected with miRNA and/or plasmid DNA using Lipofectamine 2000 (Invitrogen) following the manufacturer’s instructions, and collected 48 h later for further analysis. Biotinylated *MIRCTRL* (Exiqon #166323), *MIRLET7D* (Ambion #17010), AntiMiR *MIRLET7D-5p* (Ambion #17000), and AntiMiR-negative control (Ambion #17010) were transfected at 20 nM final concentration. Cy3-labeled *sictrl* (Ambion #17011) was used to evaluate miRNA transfection efficiency. shRNA plasmids *shEXOSC10* (Sigma #EHU082601), *shHDAC1* (EHU025841), and *shEZH2* (EHU073481) were used for loss-of-function in primary fibroblasts. Empty vectors were used as a negative control. For expression of dnEP300 we used pCMVβ-p300.DY-myc, which was a gift from Tso-Pang Yao (Addgene plasmid #30490; http://n2t.net/addgene:30490; RRID:Addgene_30490). Cells were treated with EZH2 inhibitor UNC-1999 (Sigma, #SML0778) at 2 µM final concentration for 24 h. The solvent used for dissolving these inhibitors, following manufacturer’s instruction, were used as a negative control.

### Bacterial culture

For cloning experiments, chemically competent *Escherichia coli* TOP10 (ThermoFisher Scientific) were used for plasmid transformation. TOP10 strains were grown in Luria broth (LB) at 37 °C with shaking at 180 r.p.m. for 16 h or on LB agar at 37 °C overnight.

### RNA isolation, RT, quantitative PCR, and TaqMan assay

Total RNA was isolated with Trizol (Invitrogen) and quantified using a Nanodrop Spectrophotometer (ThermoFisher Scientific, Germany). Synthesis of complementary DNA was performed using 1–2 µg total RNA and the High Capacity cDNA Reverse Transcription kit (Applied Biosystems). Quantitative real-time PCR reactions were performed using SYBR^®^ Green on the Step One plus Real-time PCR system (Applied Biosystems). Housekeeping genes *HPRT* and *GAPDH* were used to normalize gene expression. Primer pairs used for gene expression analysis are described in Supplementary Table 1. The location of the ChIP primers is displayed as UCSC Genome Browser snapshots accordingly.

Quantitative TaqMan MicroRNA Assay (Applied Biosystems) was performed according to the manufacturer’s instructions using primers and probes that specifically detect mature *MIRLET7D* (ThermoFisher Scientific #4427975 ID 002283) and *U6* control (ThermoFisher Scientific #4426961 ID Hs03910430_s1).

### miRNA pulldown

miR-Pd analysis was performed as described^25^ with minor adaptations. Ctrl and IPF primary fibroblasts were cultured in 10 cm dishes and transfected with Biotinylated hsa-*MIRLET7D*-5p-RNA (Exiqon #279787) or Biotinylated *MIRCTRL* (Exiqon #166320), at a final concentration of 20 nM using Lipofectamine 2000 (Invitrogen) following the manufacturer’s protocol. After 48 h, cells were pelleted at 270 g for 5 min. Cells were fixed with 1% glutaraldehyde (Sigma-Aldrich) to preserve RNA–Chromatin interactions. After washing three times with PBS, cell pellets were resuspended in 2 ml hypotonic cell lysis buffer (10 mM Tris-HCl (pH 7.4), 1.5 mM MgCl_2_, 10 mM KCl, 1 mM dithiothreitol (DTT), 25 mM NaF, 0.5 mM Na_3_VO_4,_ 40 μg/ml phenylmethylsulfonyl fluoride, protease inhibitor (Calbiochem), and RNase inhibitor (Promega) in Diethyl pyrocarbonate (DEPC)-treated water) on ice for 10 min and then spun down at 700 g for 10 min at 4 °C. Nuclear pellets were resuspended in 300 μl nuclear lysis buffer (50 mM Tris-HCl (pH 7.4), 170 mM NaCl, 20% glycerol, 15 mM EDTA, 0.1% (v/v) Triton X-100, 0.2 mM DTT, 20 mM NaF, 20 mM Na_3_VO_4_, 40 μg/ml phenylmethylsulfonyl fluoride, protease inhibitor, and RNase inhibitor in DEPC-treated water). Nuclear fraction was sonicated (Bioruptor NextGen, Diagenode) at high amplitude for 10 cycles of 30 s (on/off) pulses. M280 streptavidin magnetic beads (ThermoFisher Scientific) were blocked for 1 h at 4 °C in blocking buffer (10 mM Tris-HCl pH 6.5, 1 mM EDTA, 1 mg/ml yeast tRNA, and 1 mg/ml bovine serum albumin (BSA)) and washed twice with 1 ml washing buffer (10 mM Tris-HCl pH 7.0, 1 mM EDTA, 0.5 M NaCl, 0.1% (v/v) Triton X-100, RNAase inhibitor, and protease inhibitor). Beads were resuspended in 0.5 ml washing buffer. The nuclear extract was then added to the beads and incubated for 1 h at 4 °C with slow rotation. The beads were then washed five times with 1 ml washing buffer. RNA bound to the beads (pulled down RNA) and from 10% of the extract (input RNA) was isolated using Trizol reagent LS (Invitrogen) after DNase1 (NEB) treatment. The level of mRNA and ncRNA in the Biotinylated *MIRLET7D-5p* or Biotinylated *MIRCTRL* pulldown was quantified by qRT-PCR.

For miRNA–protein interaction analysis, nuclear protein extract after DNase1 treatment incubated with 500 pMol Biotinylated *MIRLET7D-5p* or Biotinylated *MIRCTRL* for 1 h with shaking at 4 °C. M280 streptavidin magnetic beads (ThermoFisher Scientific) were used for pulldown. Protein bound to the beads (miR-Pd) was incubated with 30 μl 2× SDS samples loading buffer, boiled at 95 °C for 5 min, spun down, and loaded on SDS-polyacrylamide gel electrophoresis (PAGE) for WB analysis.

### Chromatin RNA immunoprecipitation

Ch-RIP analysis was performed as described^54^ with minor adaptations. Briefly, cells were crosslinked by 1% formaldehyde for 10 min, lysed, and sonicated with Diagenode Bioruptor to disrupt and fragment genomic DNA. After centrifugation, the soluble chromatin was immunoprecipitated using antibodies specific against EXOSC10 (Abcam, #50558, dilution 1:1000), C1D (Aviva, #ARP50534_P050, dilution 1:200), EXOSC5 (Abcam, #69699, dilution 1:500), EXOSC1 (Abcam, #181167, dilution 1:1000), CTNNB (Abcam, #7302, dilution 1:1000), and IgG as a control (Santa Cruz, #sc-2027). Precipitated chromatin complexes were removed from the beads by incubating with 50 μl of 1% SDS with 0.1 M NaHCO_3_ for 15 min, vortexing every 5 min, and followed by treatment with DNase1. Reverse crosslinked immunoprecipitated materials were used for RNA isolation by Trizol (Invitrogen). Isolated RNA was subjected to cDNA synthesis and further for TaqMan assay.

### ChIP and sequential ChIP analysis

ChIP analysis was performed as described earlier^17^ with minor adaptations. Briefly, cells were crosslinked by 1% formaldehyde for 10 min, lysed, and sonicated with Diagenode Bioruptor to an average DNA length of 500–600 bp. After centrifugation, the soluble chromatin was immunoprecipitated using specific antibodies anti-EXOSC10 (Abcam, #ab50558), anti-EXOSC5 (Abcam, #69699), anti-EXOSC1 (Abcam, #181167), anti-CTNNB (Abcam, #7302), anti-EZH2 (Abcam, #ab3748), anti-H3K27me3 (Millipore, #07–449), anti-H3 (Abcam, #1791), anti-H3K56ac (Epigentek, #A-4026–050), anti-H3K27ac (Abcam, #ab4729), anti-HDAC1 (Abcam, #ab7028), anti-HDAC2 (Abcam, #ab16032), anti-POLII (Abcam, #ab5408, anti-EP300 (Abcam, #59240), anti-actEP300 (ThermoFisher, PA5-64531), anti-actEP300 (Biorbyt, #34557), anti-inaEP300 (Santa Cruz, #130210), anti-AcK (Cell Signaling, #9441 S)), anti-PhS (Millipore, #A-570-05-1000 × ), anti-IgG (Santa Cruz, #sc-2027), and anti-IgG (Santa Cruz, #sc-2025). Precipitated chromatin complexes were removed from the beads by incubating with 50 μl of 1% SDS, 0.1 M NaHCO_3_, for 30 min, while vortexing every 5 min. Reverse crosslinked immunoprecipitated chromatin was subjected to quantitative PCR.

Sequential ChIP (ChIP–reChIP) was performed as described earlier^17^ with slight modifications. Briefly, re-ChIP was performed using specific antibodies anti-HDAC1 (Abcam, #ab7028), anti-HDAC2 (Abcam, #ab16032), anti-AcK (Cell Signaling, #9441 S)), anti-PhS (Millipore, #A-570-05-1000×), anti-IgG (Santa Cruz, #sc-2027), and anti-IgG (Santa Cruz, #sc-2025). For re-ChIP experiments, complexes were eluted in 25 µl 10 mM DTT by incubation at 37 °C for 30 min. After centrifugation, the supernatant was diluted 20 times with re-ChIP buffer (1% Triton X-100, 2 mM EDTA, 150 mM NaCl, 20 mM Tris-HCl pH 8.0) and subjected again to the ChIP procedure. Primer pairs used for gene promoter analysis are described in Supplementary Table 1.

### Sucrose gradient ultracentrifugation

Sucrose gradients (5–40%) were prepared in native nuclear lysis buffer (0.2% IPGAL, 100 mM NaCl, 50 mM Tris-HCl pH 7.0, 1 mM EDTA, 0.2 mM DTT, 20 mM NaF, 20 mM Na_3_VO_4_, 40 μg/ml phenylmethylsulfonyl fluoride, protease inhibitor, and RNase inhibitor in DEPC-treated water) in polyallomer centrifuge tube (Beckman). Nuclear Ctrl and IPF extracts were loaded in 5 ml 5–40% sucrose gradients and centrifuged for 16 h 30 min at 132,000 × *g* in a SW50.1 ultracentrifuge rotor (Beckman). Following centrifugation, ten fractions (500 µl each) were collected manually from the bottom. Later, these fractions were used for WB analysis and RNA isolation using Trizol (Invitrogen) for TaqMan assay as described above.

### Co-IP and WB analysis

Co-IP was performed as described^17^ with minor adaptations. A total of 5 × 10^7^ cells were collected and washed three times in cold PBS, spun down at 270 g for 10 min at 4 °C. Cells were incubated in 2 ml of hypotonic cell lysis buffer (10 mM (pH 7.4) Tris-HCl, 1.5 mM MgCl_2_, 10 mM KCl, 1 mM DTT, 25 mM NaF, 0.5 mM Na_3_VO_4,_ 40 μg/ml phenylmethylsulfonyl fluoride and protease inhibitor (Calbiochem)) on ice for 10 min and then spun down at 700 g for 10 min at 4 °C. Nuclear pellets were resuspended in 300 μl nuclear lysis buffer (50 mM Tris-HCl (pH 7.4), 170 mM NaCl, 20% glycerol, 15 mM EDTA, 0.1% (v/v) Triton X-100, 0.2 mM DTT, 20 mM NaF, 20 mM Na_3_VO_4_, 40 μg/ml phenylmethylsulfonyl fluoride and protease inhibitor). Pre-cleared nuclear protein lysates (500 μg) were incubated with the appropriate antibodies on ice for 2 h and then 30 μl protein-G-sepharose beads (GE Healthcare; equilibrated once in 10 ml water and three times in washing buffer) were added and incubated overnight at 4 °C. Beads were collected and washed five times with 500 μl ice-cold washing buffer. Thirty microliters of 2× SDS sample loading buffer was added to beads, boiled at 95 °C for 5 min, spun down, and loaded on SDS-PAGE for WB analysis. IP and WB analyses were performed using specific antibodies anti-EXOSC10 (Abcam, #ab50558, dilution 1:1000), anti-EXOSC5 (Abcam, #69699, dilution 1:500), anti-EXOSC1 (Abcam, #181167, dilution 1:1000), anti-CTNNB (Abcam, #7302, dilution 1:1000), anti-EZH2 (Abcam, #ab3748, dilution 1:1000), anti-SUV39H1 (Active Motif, #39785, dilution 1:500), anti-H3 (Abcam, #1791, dilution 1:1000), anti-H3Ac (Abcam, #ab47915, dilution 1:1000), anti-C1D (Aviva, #ARP50534_P050, dilution 1:200), anti-HDAC1 (Abcam, #ab7028, dilution 1:1000), anti-HDAC2 (Abcam, #ab16032, dilution 1:1000), anti-FLAG M2 (Sigma, #F1804, dilution 1:1000), anti-FN1 (Millipore, #AB2033, dilution 1:1000), anti-COL1A1 (Sigma, #C2456, dilution 1:500), anti-ACTA2 (Sigma, #A5228, dilution 1:500), anti-GAPDH (Sigma, #G8795, dilution 1:1000), anti-HA (Santa Cruz, #sc-805, dilution 1:1000), anti-MYC-tag (Abcam, #9132, dilution 1:1000), anti-EP300 (Abcam, #59240, dilution 1:1000), anti-actEP300 (ThermoFisher, PA5-64531, dilution 1:1000), anti-inaEP300 (Santa Cruz, #130210, dilution 1:500), and anti-VIM (Cell Signaling, #5741, dilution 1:500). Immunoreactive proteins were visualized with the corresponding horseradish peroxidase-conjugated secondary antibodies (Santa Cruz) using the Super Signal West Femto detection solutions (ThermoFisher Scientific). Signals were detected and analyzed with Luminescent Image Analyzer (Las 4000, Fujifilm). Protein concentrations were determined using Bradford substrate (Sigma).

### miRNA fluorescence in situ hybridization

miRNA-FISH was performed as described earlier^25^ with minor adaptations. Briefly, primary cells were fixed with 4% paraformaldehyde (PFA), dehydrated with 70% ethanol, and incubated with pre-hybridization buffer (50% formamide, 5× SSC, 5× Denhardt’s solution, 200 µg/ml yeast RNA, 500 µg/ml salmon sperm DNA, and 2% Roche blocking reagent in DEPC-treated water). After 4 h, pre-hybridization buffer was replaced with denaturizing hybridization buffer (10% CHAPS, 20% Tween, 50% formamide, 5× SSC, 5× Denhardt’s, 200 µg/ml yeast RNA, 500 µg/ml salmon sperm DNA, and 2% Roche blocking reagent in DEPC-treated water) containing biotin and/or digoxin-labeled Locked Nucleic Acid (LNA^TM^) probes (Exiqon) specific to mature *Mirlet7d*, to a final concentration of 20 pM and incubated at 55 °C overnight. Next day, cells were briefly washed with 5% SSC buffer pre-warmed to 60 °C and then incubated with 0.2% SSC at 60 °C for 1 h. Later, cells were incubated with B1 solution (0.1 M Tris pH 7.5, 0.15 M NaCl) at room temperature (RT) for 10 min. B1 solution was then replaced with blocking solution (10% FCS, 0.1 M Tris pH 7.5, 0.15 M NaCl) and incubated for 1 h at RT. Cells were then incubated with fluorescein isothiocyanate-labeled anti-Biotin (Abcam, #53469) and/or Alexa fluor 647-labeled mouse anti-Digoxin (Dianova) antibodies overnight at 4 °C. In order to couple mature *Mirlet7d*-FISH with EXOSC10 immunostaining, after completion of miRNA-FISH procedure, cells were incubated with mouse anti-EXOSC10 (Santa Cruz, #374595, dilution 1:500) antibody overnight at 4 °C followed by Alexa Fluor 594-tagged anti-mouse secondary antibody (Invitrogen, Germany, dilution 1:2000) and 4′,6-diamidino-2-phenylindole (DAPI) (Sigma, Germany) for 1 h at RT. DAPI was used as nuclear dye. Cells were examined with a confocal microscope (Zeiss).

### Immunohistochemistry and PLA assay

Immunostaining was performed as previously described^17^. Briefly, cells were grown on coverslips, fixed with 4% PFA for 10 min at RT, and permeabilized with 0.4% Triton X-100/1× PBS for 10 min at RT. During immunostaining procedure, all incubations and washes were performed with histobuffer containing 3% BSA and 0.2% Triton X-100 in 1× PBS, pH 7.4. Nonspecific binding was blocked by incubating with donkey serum and histobuffer (1:1 (v/v) ratio) for 1 h. Cells were then incubated with primary and then secondary antibodies for 1 h followed by nuclear staining. Immunostaining of cells were examined with a confocal microscope (Zeiss). Antibodies used were specific anti-ACTA2 (Sigma, #A5228, dilution 1:500), anti-EP300 (Abcam, #59240, dilution 1:500), anti-actEP300 (Biorbyt, #34557, dilution 1:500), and anti-inaEP300 (Santa Cruz, #130210, dilution 1:500). Alexa 488- or Alexa 594-tagged secondary antibodies (Invitrogen, Germany, dilution 1:2000) were used. DAPI (Sigma, Germany) was used as nuclear dye.

Paraffin-embedded lung tissue sections (3 μm thick) were deparaffinized in xylene and rehydrated in a graded ethanol series to PBS (pH 7.2). Antigen retrieval was performed by pressure cooking in citrate buffer (pH 6.0) for 15 min. Double immunofluorescence staining was performed with primary antibodies anti-ACTA2 (Sigma, #A5228, dilution 1:500), anti-EP300 (Abcam, #59240, dilution 1:500), anti-actEP300 (Biorbyt, #34557, dilution 1:500), and anti-inaEP300 (Santa Cruz, #130210, dilution 1:500). After overnight incubation with specific primary antibodies, slides were washed and incubated with the respective secondary antibodies, Alexa 488- and Alexa 555-conjugated goat anti-rabbit IgG (dilution 1:1000, Molecular Probes, Eugene, OR) for 1 h. All sections were counterstained with nuclear DAPI (1:1000) and mounted with fluorescent mounting medium (Dako).

For PLA assay, cells were grown on coverslips, fixed, permeabilized, blocked, and incubated with primary antibodies as in immunostaining discussed above. After primary antibody incubation, the PLA was performed using the Duolink in situ Fluorescence Kit (Sigma, #DUO92101-1KT) according to the manufacturer’s instructions. The protein–protein interactions were analyzed by using confocal microscope (Zeiss). Primary antibody combination was goat anti-HDAC1 (Santa Cruz, #6298, dilution 1:500) and rabbit anti-AcK (Cell Signaling, #9441 S, dilution 1:500). DAPI (Sigma, Germany) was used as nuclear dye.

### HDAC activity assay

Assays were performed using the colorimetric HDAC activity assay from Bio Vision (Bio Vision Research Products, Mountain View, CA, USA) according to the manufacturer’s instructions. Briefly, 50 μg of nuclear extracts from Ctrl and IPF primary fibroblasts were diluted in 85 μl of ddH_2_O; then, 10 μl of 10× HDAC assay buffer were added, followed by addition of 5 μl of the colorimetric substrate; samples were incubated at 37° for 1 h. Subsequently, the reaction was stopped by adding 10 μl of lysine developer and left for additional 30 min at 37 °C. Samples were then read in an enzyme-linked immunosorbent assay plate reader at 400 nm. HDAC activity was expressed as relative OD values per μg of protein sample. The kit contains negative and positive controls that consist of nuclear extract of HeLa treated or not with Trichostatin A, respectively.

### HDAC1-specific activity assay

Assays were performed using the HDAC1 fluorogenic assay kit from BPS Bioscience (Catalog #50061) according to the manufacturer’s instructions. In this assay, recombinant, full-length HDAC1 protein as a positive control and nuclear and cytoplasmic extracts from Ctrl and IPF primary fibroblasts were incubated with fluorophore-conjugated substrates, at a concentration equivalent to the substrate Km (6 μM for HDAC1). Reactions were performed in assay buffer (50 mM HEPES, 100 mM KCl, 0.001% (v/v) Tween-20, 0.05% (w/v) BSA, 200 μM Tris (2-carboxyethyl)phosphine (TCEP) pH 7.4) and followed for fluorogenic release of 7-amino-4-methylcoumarin from substrate upon deacetylase and trypsin enzymatic activity. Fluorescence measurements were obtained at 470 nm approximately every 5 min using a multilabel plate reader. Data were analyzed on a plate-by-plate basis for the linear range of fluorescence over time. The kit contains Trichostatin A as negative controls.

### Migration and proliferation assays

Ctrl and IPF primary fibroblasts treated with EP300inh were grown till 90–95% confluence in a six-well plate. Monolayers were scratched with a 200 μl pipette tip (Biozym), washed to remove debris, and cultured to facilitate cell migration. Images were collected at the indicated time points using a ×5 objective on a phase-contrast microscope (Leica DMI3000 B) to quantify cell migration distance at 12 h after scratch as computed by D12h = L12h − L0h; where D12h and L12h are the net cell migration distance and the cell position at the 12 h time point, respectively. L0h is the original position^55^. Lung fibroblasts, to be assessed for cellular proliferation, were cultured either in 96‐ or 48‐well plates. Fibroblast proliferation was determined using colorimetric bromodeoxyuridine incorporation assay kit (Roche, Germany) according to the manufacturer’s instructions. Absorbance was measured at 370 nm with reference at 492 nm in a plate reader (TECAN, Germany). Depending on the experiment, proliferation of cells was plotted either as the difference of absorbance at 370 and 492 nm (A370 nm–A492 nm) or as a percentage of absorbance compared with control cell absorbance.

### Fibrosis score and collagen content

Fibrosis score was assessed using the Ashcroft score as previously described^56^; the grade (from 0 to 8) of fibrotic changes in histological lung samples were scaled numerically. Sections (4 μm) of the left lobe of mice lungs were stained with hematoxylin and eosin. Whole sections were scanned with tenfold magnification using Leica DM6000B microscope (Leica, Germany). This resulted in around 25–40 pictures per mouse lung. Pictures from each mouse lung were graded, averaged, and depicted in a graph. Whole‐lung lobe pictures were scanned with Nanozoomer 2.0 HT (Hamamatsu, Japan). Collagen content was assessed by Sirius red staining of lung sections and the area of collagen was measured using Leica image software (Leica Microsystems, Germany) according to the standard protocol.

### Collagen assay

Total collagen content was determined using the Sircol Collagen Assay kit (Biocolor, Belfast, Northern Ireland). One lobe from the right lung was taken for analysis and the weight determined. Equal amounts of protein lysates were added to 1 ml of Sircol dye reagent, followed by 30 min of mixing. After centrifugation at 10,000 × *g* for 10 min, the supernatant was carefully aspirated and 1 ml of Alkali reagent was added. Samples and collagen standards were then read at 540 nm on a spectrophotometer (Bio-Rad). Collagen concentrations were calculated using a standard curve generated by using acid-soluble type 1 collagen.

### Hydroxyproline measurements

Hydroxyproline levels in human lung fibroblasts and lung tissue were determined using the QuickZyme Hydroxyproline Assay kit (Quickzyme Biosciences, Leiden, the Netherlands). The cells and lung tissue were separately homogenized in 1 ml 6 N HCl with a Precellys tissue homogenizer (2 × 20 s, 3,800 × *g*). The homogenate was then hydrolyzed at 90 °C for 24 h. After centrifugation at 13,000 × *g* for 10 min, 100 μl from the supernatant was taken and diluted 1:2 with 4 N HCl. Thirty-five microliters of this working dilution was transferred to a 96-well plate. Likewise, a hydroxyproline standard (12.5–300 μM) was prepared in 4 N HCl and transferred to the microtiter plate. Following addition of 75 μl of a chloramine T-containing assay buffer, samples were oxidized for 20 min at RT. The detection reagent containing *p*-dimethylaminobenzaldehyde was prepared according to the manufacturer’s instruction and 75 μl added to the wells. After incubation at 60 °C for 1 h, the absorbance was read at 570 nm with a microtiter plate reader (Infinite M200 Pro, Tecan, Crailsheim, Germany) and the hydroxyproline concentration in the sample was calculated from the standard curve and related to the employed amount of lung tissue. The hydroxyproline content in lung tissue is given as μg hydroxyproline per mg lung tissue.

### Experiments with mouse PCLS

PCLS were prepared from male, 12–16 weeks old C57BL/6 mice. Animals were humanely killed with mixture of ketamine and xylazine. A skin incision was performed on the ventral side of the neck and the trachea was exposed. A small incision was made on the trachea to allow insertion of an 18G catheter. Once the abdominal and chest cavities were opened, the collapsed lung lobes were infused with 1.5 ml of warmed (37 °C) 1.5% agarose in Dulbecco’s modified Eagle’s medium (DMEM) (Gibco) through the inserted catheter. The agarose infusion was followed by 0.5 ml of air to clear the airways of agarose. The whole mouse was placed at 4 °C for 10–15 min to allow the agarose to solidify. The lungs were then carefully dissected out of the chest cavity and kept in cold DMEM prior to slicing. After the selected lobes were separated, 8 mm lung tissue cores were prepared and sectioned into 250 µm-thick slices using a Krumdieck microtome (Alabama Research and Development, Munford, AL). PCLS were sorted serially during the slicing process, to ensure that every exposure group consisted of adjacent PCLS from throughout (distal, middle, and proximal regions) the core. After slicing, the sorted PCLS were incubated (3 PCLS per well in a 24-well plate) overnight in DMEM/Ham’s F-12 Medium (Corning, Corning, New York) supplemented with 0.05 mg/ml Kanamycin (Sigma-Aldrich, St. Louis, MO), 1% Gibco Antibiotic-Antimycotic, containing 10,000 units/ml of penicillin, 10 mg/ml of streptomycin, and 25 µg/ml of Amphotericin B (Life Technologies) at 37 °C.

### Experiments with human PCLS

PCLS were prepared from tumor-free lung explants from patients who underwent lung resection for cancer at KRH Hospital Siloah-Oststadt-Heidehaus or the Hanover Medical School (both Hanover, Germany). Tissue was processed immediately within 1 day of resection as described before^57^. Briefly, human lung lobes were cannulated with a flexible catheter and the selected lung segments were inflated with warm (37 °C) low-melting agarose (1.5%) dissolved in DMEM Nutrient Mixture F-12 Ham supplemented with l-glutamine, 15 mM HEPES without phenol red, pH 7.2–7.4 (Sigma-Aldrich, Hamburg, Germany), 100 U/mL penicillin, and 100 µg/mL streptomycin (both from Biochrom, Berlin, Germany). After polymerization of the agarose solution on ice, tissue cores of a diameter of 8 mm were prepared using a sharp rotating metal tube. Subsequently, the cores were sliced into 300–350 µm thin slices in DMEM using a Krumdieck tissue slicer (Alabama Research and Development, Munford, AL). PCLS were washed 3× for 30 min in DMEM and used for experiments. Viability of the tissue was assessed by a LDH Cytotoxicity Detection Kit (Roche, Mannheim, Germany) according to the manufacturer’s instruction. Human PCLS were treated with Ctrl or pro-fibrotic cocktail as previously described^58^.

### RNA-seq and data analysis

RNA-seq of nuclear extracts from Ctrl and IPF primary fibroblasts was performed by Single End sequencing using Nextseq500 (Illumina) sequencer with V2 chemistry. RiboGone™ - Mammalian (Clontech) was used to deplete ribosomal RNA from the samples. SMARTer® Universal Low Input RNA Kit for Sequencing (Clontech) was used for pre-amplification before library preparation. Library preparation was performed using the Low Input Libray Prep Kit (Clontech) following the manufacturer’s instructions.

Raw reads were visualized by FastQC^59^ to determine the quality of the sequencing. Trimming was performed using trimmomatic^60^ with the following parameters LEADING:3 TRAILING:3 SLIDINGWINDOW:4:15 MINLEN:40 CROP:45 HEADCROP:5.

High quality reads were mapped to the NONCODE V4.0 (http://www.noncode.org/download.php) for the ncRNAs or hg19 genome using bowtie2^61^ with -D 15 -R 3 -N 0 -L 20 -i S, 1, 0.56 -p8. After mapping, the sam files were converted to bam format by using samtools view (-Sb) followed by samtools sort. The sorted bam files were further converted to fastq files with SamToFastq.jar from picard-tools-1.119 (https://sourceforge.net/projects/picard/files/picard-tools/1.119/) and remapped to hg19 (UCSC reference genome). The tag libraries were created by using MakeTaglibrary from HOMER^62^ (-format sam). The makeUCSCfile command was used for visualizing the mapped sequence tag density after normalizing the samples to ten million. For the ncRNAs and for protein coding RNAs, samples were quantified by using analyzeRepeats.pl (http://homer.salk.edu/homer/ngs/analyzeRNA.html) with the parameters (analyzeRepeats.pl database.gtf hg19 -count genes -strand both and -rpkm; reads per kilobase per millions mapped). The NONCODE_V.4.gtf file was used to quantify ncRNAs. KNIME 2.9.1 platform (Konstanz, Germany) was used to filter enriched ncRNAs and coding RNAs in the IPF compared with the Ctrl, by using the column filter, numeric binner, and row filter nodes.

UCSC genome browser was used to visualize Ctrl and IPF nuclear transcripts. Two scales were used for ncRNA and mRNA visualization.

### Reporting summary

Further information on research design is available in the Nature Research Reporting Summary linked to this article.

## Supplementary information

Supplementary Information

Reporting Summary

Source Data

## Data Availability

Sequencing data of nuclear RNA in Ctrl and IPF patients have been deposited in NCBI’s Gene Expression Omnibus^63^ and is accessible through GEO Series with accession number GSE116086. RNA-seq data from total lung homogenates was retrieved through the GEO Series accession number GSE52463. The source data for all the plots and uncropped pictures of all the blots presented in the main and Supplementary Figures are provided as a Source Data file. Please direct any request for further information or reagents to Lead Contact Guillermo Barreto (guillermo.barreto@mpi-bn.mpg.de), LOEWE Research Group Lung Cancer Epigenetic, Max-Planck-Institute for Heart and Lung Research, Parkstrasse 1, 61231 Bad Nauheim, Germany.
