# Supplementary material for: Inactivation of nuclear histone deacetylases by EP300 disrupts the MiCEE complex in idiopathic pulmonary fibrosis
Source: Nat Commun. 2019 May 20;10:2229. doi: 10.1038/s41467-019-10066-7 (PMC6527704; doi:10.1038/s41467-019-10066-7)
Supplement: Supplementary file 1 — Supplementary Information [file 41467_2019_10066_MOESM1_ESM.docx]

**Supplementary Information for**

**Inactivation of nuclear histone deacetylases by EP300 disrupts the MiCEE complex in Idiopathic Pulmonary Fibrosis**

Karla Rubio^1^, Indrabahadur Singh^1, 2, *^, Stephanie Dobersch^1^, Pouya Sarvari^3^, Stefan Günther^4^, Julio Cordero^1, 5, 6^, Aditi Mehta^1, 7^, Lukasz Wujak^8^, Hector Cabrera-Fuentes^8, 9, 10,11,22^, Cho-Ming Chao^12, 13, 14, 15, 16^, Peter Braubach^15, 17, 18^ ,Saverio Bellusci^10, 12, 13, 14, 15^, Werner Seeger^3, 14, 16, 19^, Andreas Günther ^14, 15, 19, 20^, Klaus T Preissner^8, 10, 14^, Malgorzata Wygrecka^8, 14, 15^, Rajkumar Savai^3, 14, 15^, Dulce Papy-Garcia^21^, Gergana Dobreva^5, 6^, Mathias Heikenwalder^2^, Soni Savai-Pullamsetti^3, 14, 15^, Thomas Braun^4, 14^ and Guillermo Barreto^1, 10, 14, 15, 21, *^

*Correspondence to: [guillermo.barreto@mpi-bn.mpg.de](mailto:guillermo.barreto@mpi-bn.mpg.de); i.singh@dkfz-heidelberg.de

**This PDF file includes:**

Supplementary Discussion

Supplementary Figures 1 to 7

Supplementary Table 1

Supplementary References

**Other Supplementary Materials for this manuscript include the following:**

Source Data File (separate file). Excel file containing the values and statistical summary of the complete manuscript, and theuncropped pictures of all the western blots presented in the manuscript.

Supplementary Information

Supplementary Discussion

**Nuclear histone deacetylase activity is reduced in Idiopathic Pulmonary Fibrosis**

To link the results obtained using protein extracts from primary fibroblasts with the *MIRLET7D* target genes, we analyzed by ChIP the promoters of these genes using antibodies specific for HDAC1 and H3K56Ac (acetylated Lys-56 of histone 3) (Supplementary Figure 4c). H3K56Ac is a substrate for HDAC1 and its levels are considered to be inversely proportional to HDAC1 activity ^1^. We observed increased levels of both HDAC1 and H3K56Ac at the promoter of all analyzed genes in IPF when compared to Ctrl fibroblasts, suggesting enrichment of inactive HDAC1 at the promoters. Transient transfection of mature *MIRLET7D* in IPF fibroblasts did not significantly change the levels of HDAC1 when compared to *MIRCTRL* transfected IPF fibroblasts. Interestingly, *MIRLET7D* significantly reduced H3K56Ac levels in all promoters analyzed, indicating that *MIRLET7D*-GOF was sufficient to reconstitute HDAC1 activity in IPF fibroblasts. These results were confirmed by ChIP and sequential ChIP (ChIP-reChIP) analysis of *MIRLET7D* targets using HDAC1- and AcK-specific antibodies in Ctrl and IPF fibroblasts (Supplementary Figure 4d, left). We detected in *MIRCTRL*-transfected IPF fibroblasts enrichment of inactive (AcK) HDAC1 in all promoters analyzed. *MIRLET7D* gain-of-function (GOF) in IPF fibroblasts reduced the levels of inactive (AcK) HDAC1. It has been reported that HDAC1 acts upstream of HDAC2 and inactivation of HDAC1 leads to inactivation of HDAC2 *^2^*. Thus, we analyzed *MIRLET7D* targets by ChIP-reChIP using HDAC2- and PhS-specific antibodies in Ctrl and IPF fibroblasts (Supplementary Figure 4d, right). Similar to HDAC1, we detected in *MIRCTRL*-transfected IPF fibroblasts inactive (PhS) HDAC2 in al promoters analyzed, whereas *MIRLET7D*-GOF in IPF fibroblasts reduced the levels of inactive (PhS) HDAC2. Summarizing, our results demonstrated that HDAC1 and HDAC2 are inactive in nucleus of IPF fibroblasts and *MIRLET7D*-GOF was sufficient to reconstitute their activity.


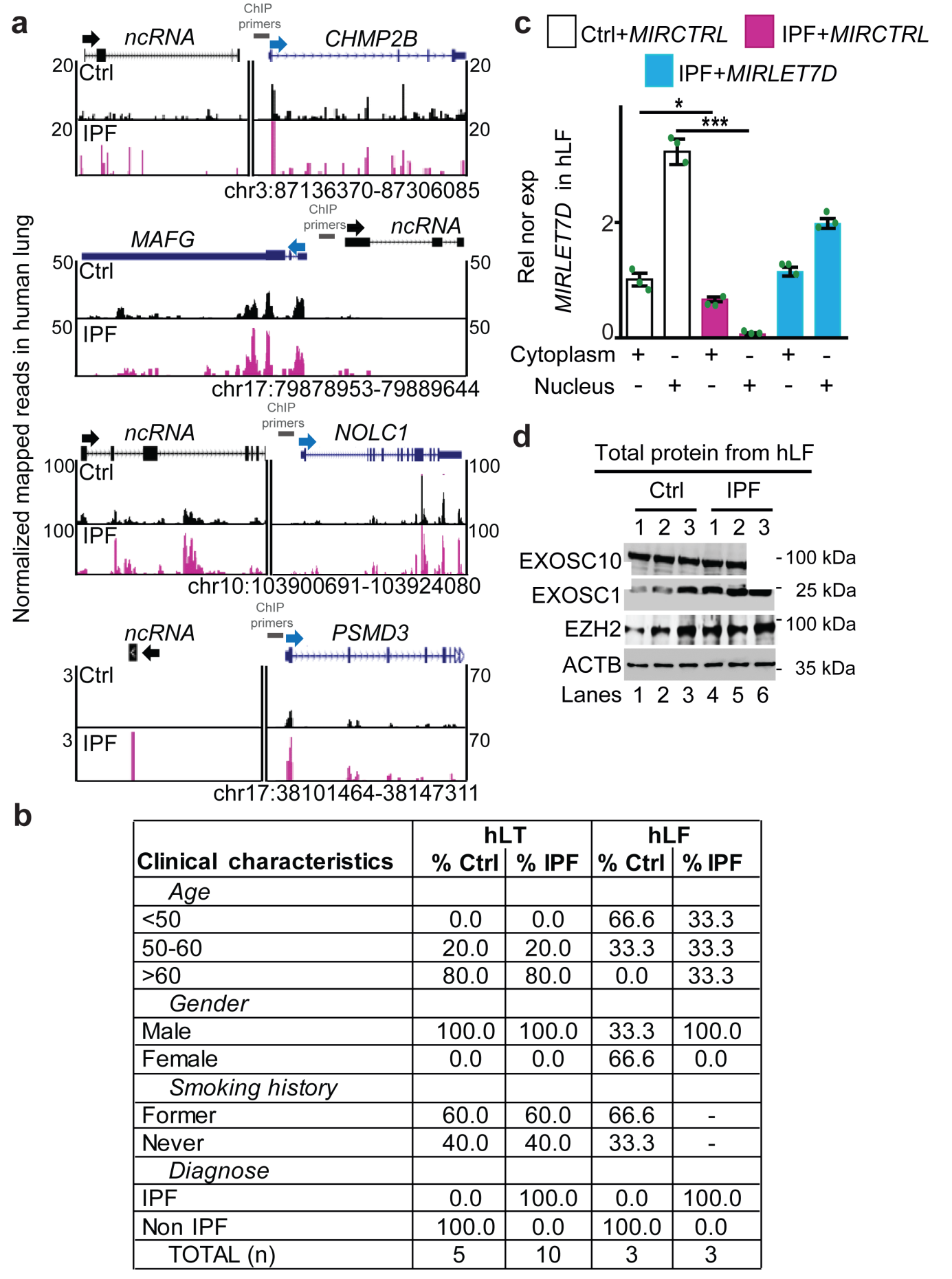


**Supplementary Figure 1.**

***MIRLET7D* target genes in human and patient characteristics.** **a**, Genome-browser visualization of representative cRNAs loci enriched in IPF (magenta) compared to Ctrl (black) by RNA-seq-based expression analysis in patients’ total lung homogenate. Images represent mapped sequence tag densities relative to the indicated loci; blue boxes, cRNA exons; black boxes, adjacent ncRNA exons; lines, introns; arrow, transcription direction. **b**, Clinical characteristics of Ctrl and IPF patients, from which samples were derived and used in the present study. Human lung tissue, hLT; primary human lung fibroblasts, hLF. **c**, Mature *MIRLET7D* specific TaqMan assay after cellular fractionation of Ctrl or IPF hLF that were transfected with *MIRCTRL* or *MIRLET7D*. Data are shown as mean (SD) (*n*=3 biologically independent experiments). Asterisks in all plots, P values after unpaired t-Test, two tailed, ****P*˂0.001; ***P*˂0.01; **P*˂0.05; ns, not significant. **d**, Western blot (WB) using the indicated antibodies and whole-cell protein extracts from Ctrl and IPF primary human lung fibroblasts (hLF) derived from three different donors, each. Source data are provided as a Source Data file.


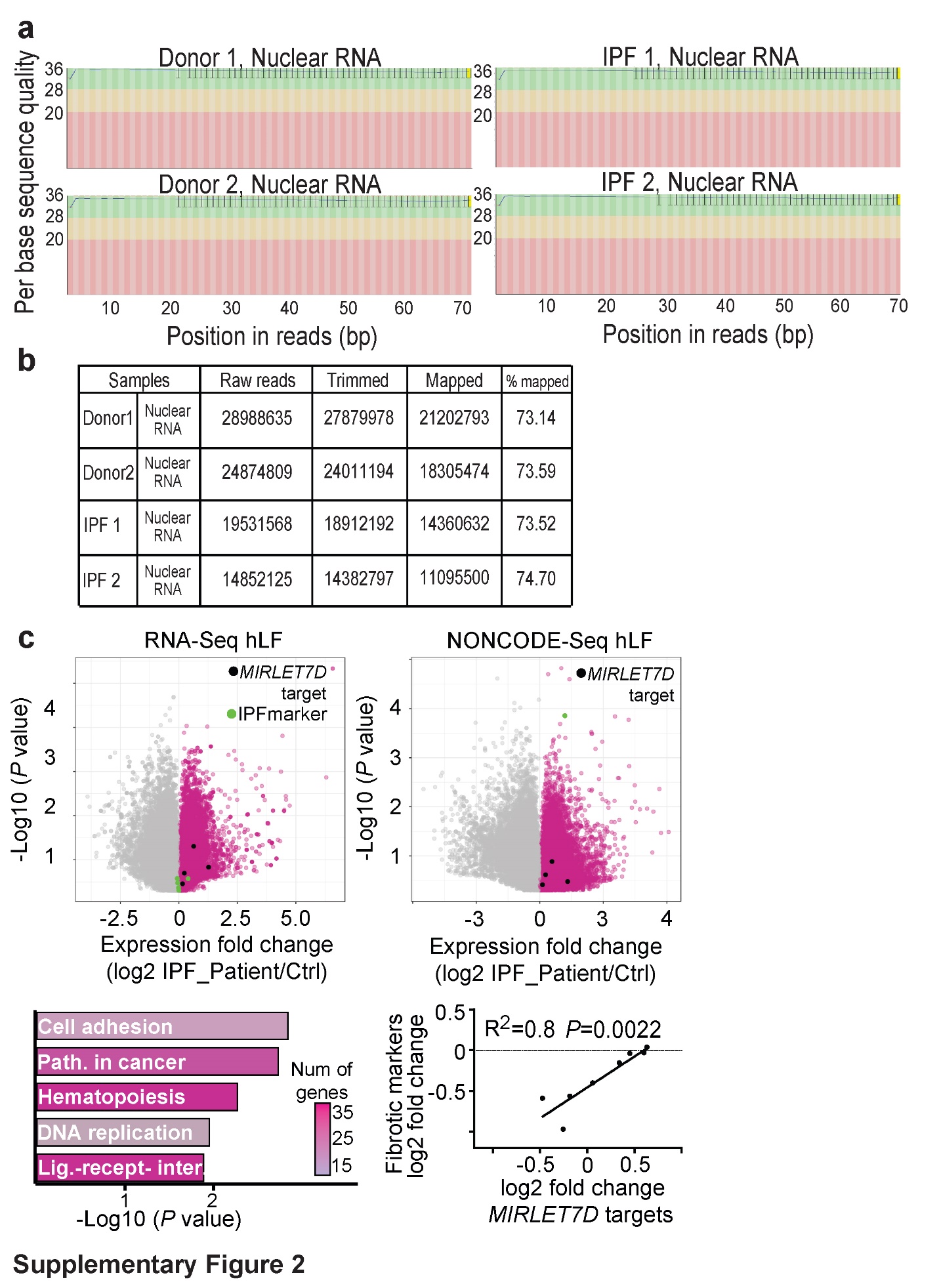


**Supplementary Figure 2.**

***MIRLET7D* ncRNA targets expression in IPF correlates with known fibrotic markers.** **a**, RNA-sequencing using the nuclear fraction of Ctrl and IPF patient-derived primary fibroblasts (accessible through GEO Series with accession number GSE116086). Phred quality score distribution over all reads in each base. The score is divided into very good quality calls (green), calls of reasonable quality (orange), and calls of poor quality (red). **b**, Description of the RNA-seq data sets supports the quality of the experiment. **c**, Top, Volcano plot representing the significance (-log10 P values after Welch´s t-Test) *versus* expression fold change (log2 expression ratios) between Ctrl and two IPF patients of coding (left) and non-coding (right) transcripts. Magenta dots show transcripts with positive log2 expression ratios. Black dots show *MIRLET7D* targets. Green dots show fibrotic markers. Bottom, KEGG-based enrichment analysis of up-regulated transcripts in IPF using DAVID bioinformatics tool and plotted by highest significance (-log10 of modified Fisher exact *P* value). Bar color represents number of genes in each group based on the legend. Path., pathways; lig., ligand; recept., receptor; inter., interaction. *Lower right*, correlation analysis between *MIRLET7D* targets and fibrotic markers by linear regression of log2 of the fold change IPF/Ctrl values.


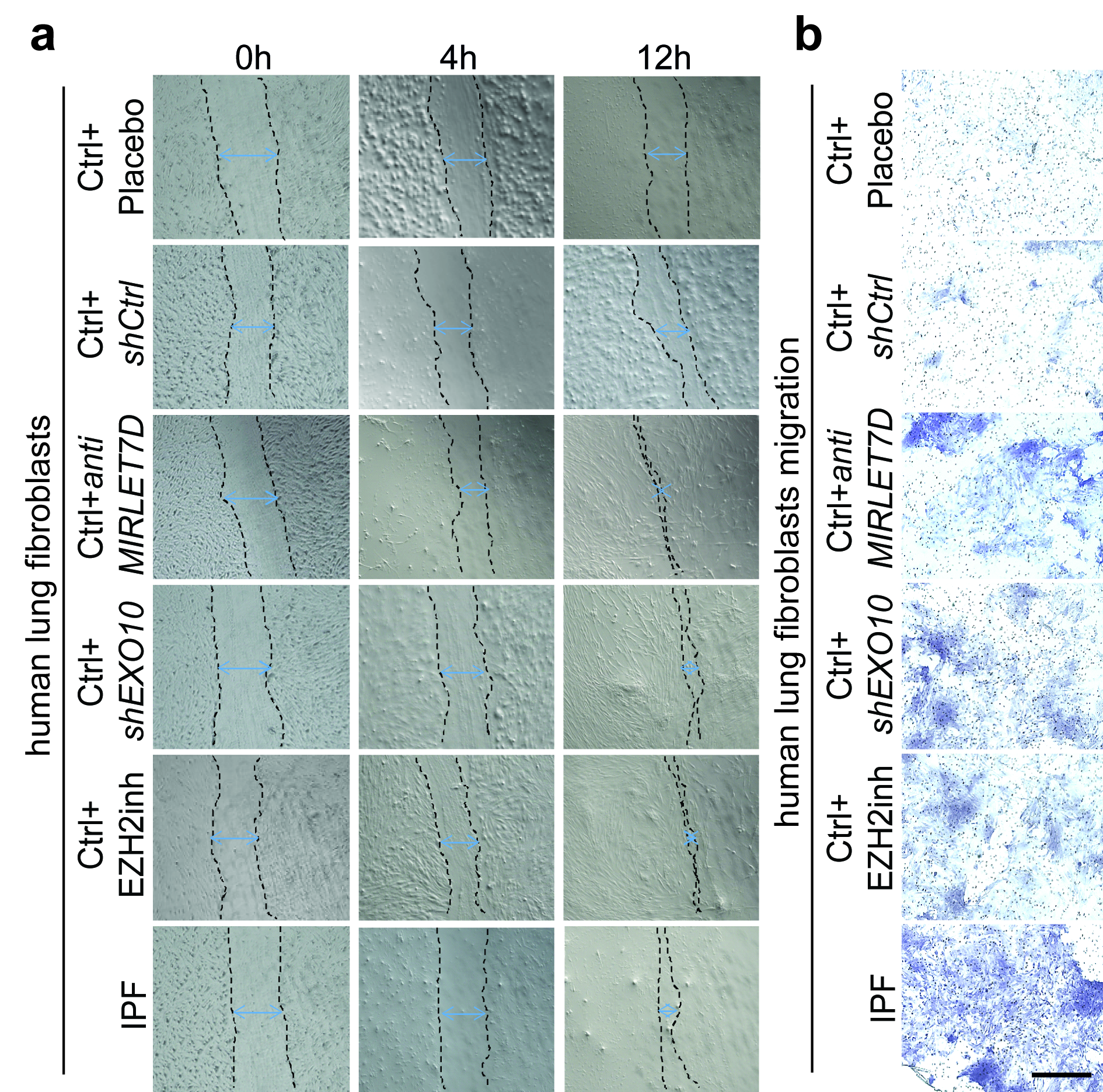


**Supplementary Figure 3.**

**Loss-of-function of MiCEE components increased fibrosis hallmarks.** **a-b**, LOF of *MIRLET7D* by antagomiR probes (*antiMIRLET7D*), EXOSC10 by short hairpin RNA construct (*shEXO10*) and EZH2 by small-molecule inhibitor UNC-1999 (EZH2inh) in Ctrl human primary lung fibroblasts (hLF) increased cell migration to similar levels as in IPF hLF. Scratch migration assays (**a**) of Ctrl or IPF hLF at 0, 4 and 12 hours after scratch (see Figure 4a). Transwell invasion assays followed by hematoxylin and eosin (H&E) stain (**b**) of Ctrl or IPF hLF (see Figure 4b). Source data are provided as a Source Data file.


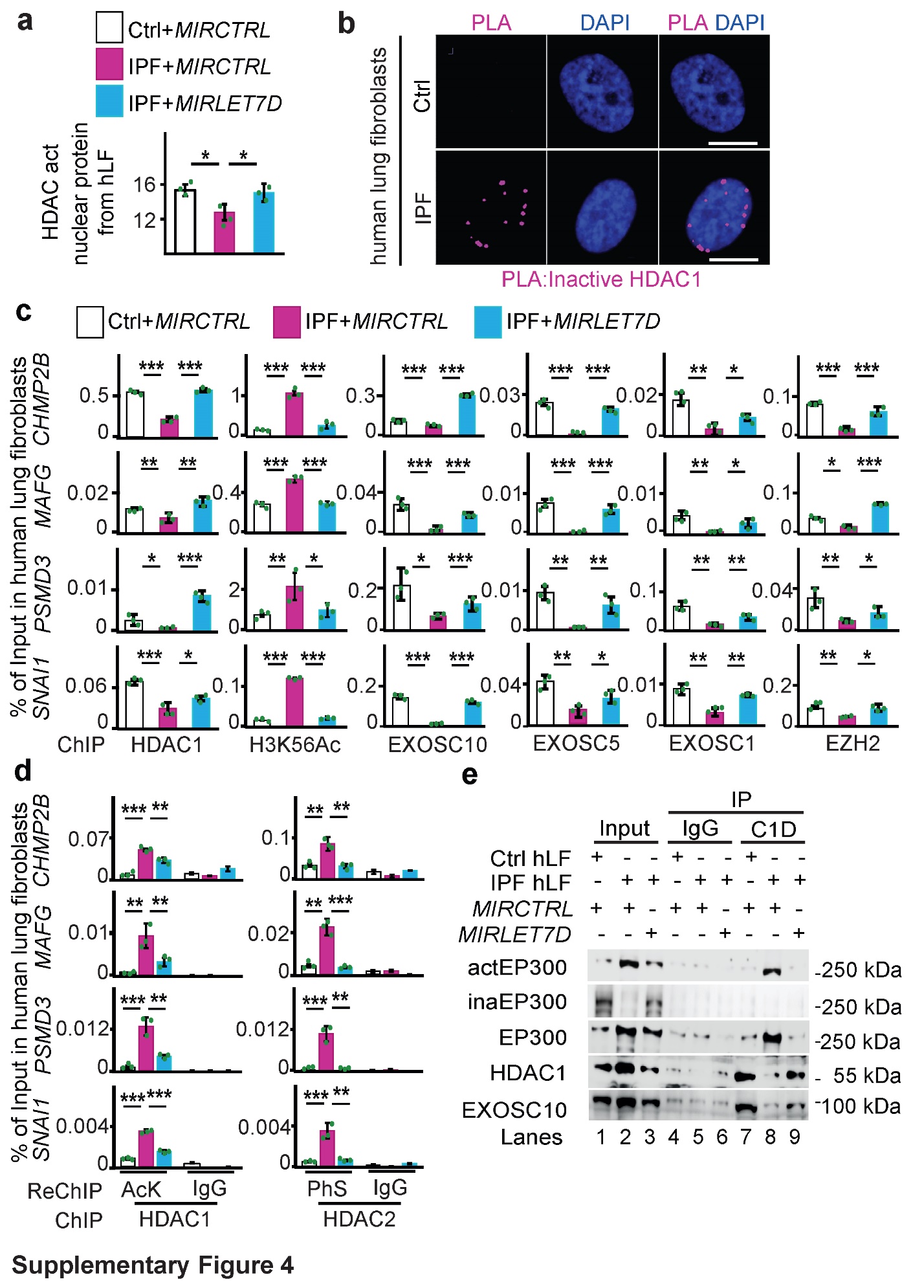


**Supplementary Figure 4.**

**Reduced nuclear histone deacetylase activity in IPF is restored by *MIRLET7D*.** **a**, HDAC activity assay using nuclear protein extracts from Ctrl or IPF primary human lung fibroblasts (hLF) that were transfected with control miRNA (*MIRCTRL*) or *MIRLET7D* probes. **b**, Confocal microscopy after proximity ligation assay using HDAC1- and AcK-specific antibodies shows acetylation-dependent HDAC1 inactivation in IPF but not in Ctrl primary fibroblasts. DAPI, nucleus. Scale bars, 20 µm. **c**, **d**, ChIP (**c**) or sequential ChIP (ChIP-reChiP, **d**) analysis of representative *MIRLET7D* target promoters using HDAC1 or H3K56ac-specific antibodies, or HDAC1 or HDAC2-specific antibodies followed by pan-acetyl-lysine (AcK) or phospho-serine (PhS)-specific antibodies respectively, and chromatin isolated from Ctrl or IPF hLF that were transfected as in **a**. IgG, immunoglobulin G (negative control). In all bar plots, data are shown as mean (SD) (*n*=3 biologically independent experiments). Asterisks in all plots, *P* values after unpaired t-Test, two tailed, ****P*˂0.001; ***P*˂0.01; **P*˂0.05; ns, not significant. **e**, WB using the indicated antibodies after Co-IP assay using immunoglobulin G (IgG; negative control) or C1D-specific antibodies to immunoprecipitate endogenous C1D from nuclear protein extracts of Ctrl or IPF hLF that were transfected as in **a**. Input, 5% of IP starting material. Source data are provided as a Source Data file.


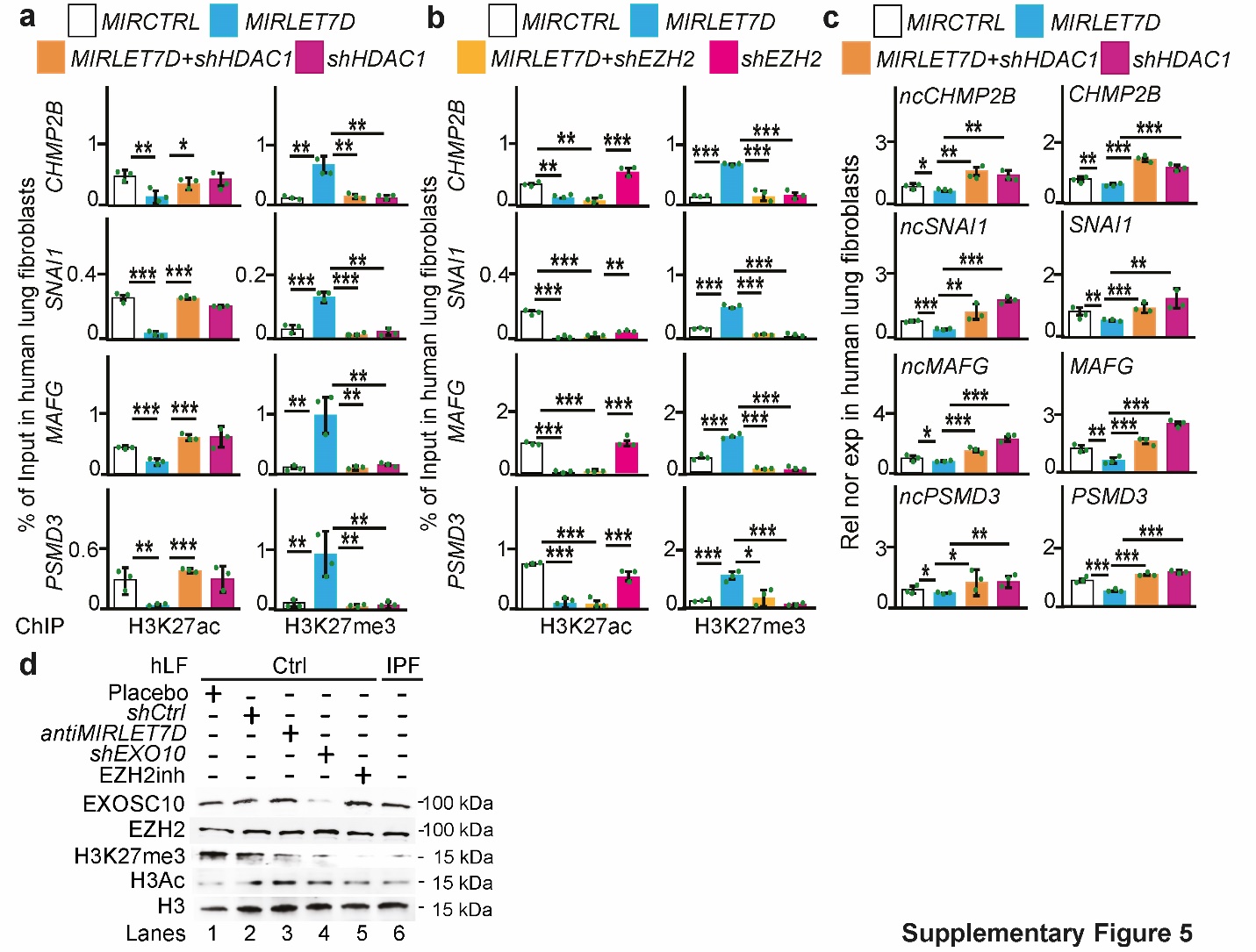


**Supplementary Figure 5.**

**HDAC1-mediated deacetylation of H3K27 is required and anticipates EZH2-mediated methylation of the same amino acid residue.** **a**, ChIP analysis of representative *MIRLET7D* target promoters *^3^* using H3K27ac or H3K27me3-specific antibodies and chromatin isolated from Ctrl human primary fibroblasts (hLF) that were transfected with *MIRCTRL*, *MIRLET7D* or/and *HDAC1*-specific *shRNA* constructs. IgG, immunoglobulin G (negative control). **b**, ChIP analysis of *MIRLET7D* target promoters using H3K27ac or H3K27me3-specific antibodies and chromatin isolated from Ctrl human primary fibroblasts (hLF) that were transfected with *MIRCTRL*, *MIRLET7D* or/and *EZH2*-specific *shRNA* constructs. IgG, immunoglobulin G (negative control). **c**, qRT-PCR-based analysis of noncoding RNA (ncRNA) and cRNA of representative nuclear *MIRLET7D* targets in Ctrl human primary fibroblasts (hLF) transfected with *MIRCTRL*, *MIRLET7D* or/and *HDAC1*-specific *shRNA* constructs. In all bar plots, data are shown as mean (SD) (*n*=3 biologically independent experiments). Asterisks in all plots, *P* values after unpaired t-Test, two tailed, ****P*˂0.001; ***P*˂0.01; **P*˂0.05. **d**, Western blot of whole-cell protein extracts from Ctrl or IPF human primary lung fibroblasts (hLF) after loss-of-function of *MIRLET7D* by antagomiR probes (*antiMIRLET7D*), *EXOSC10* by short hairpin RNA construct (*shEXO10*) and EZH2 by small-molecule inhibitor UNC-1999 (EZH2inh) using the indicated antibodies. See Figure 4. Source data are provided as a Source Data file.


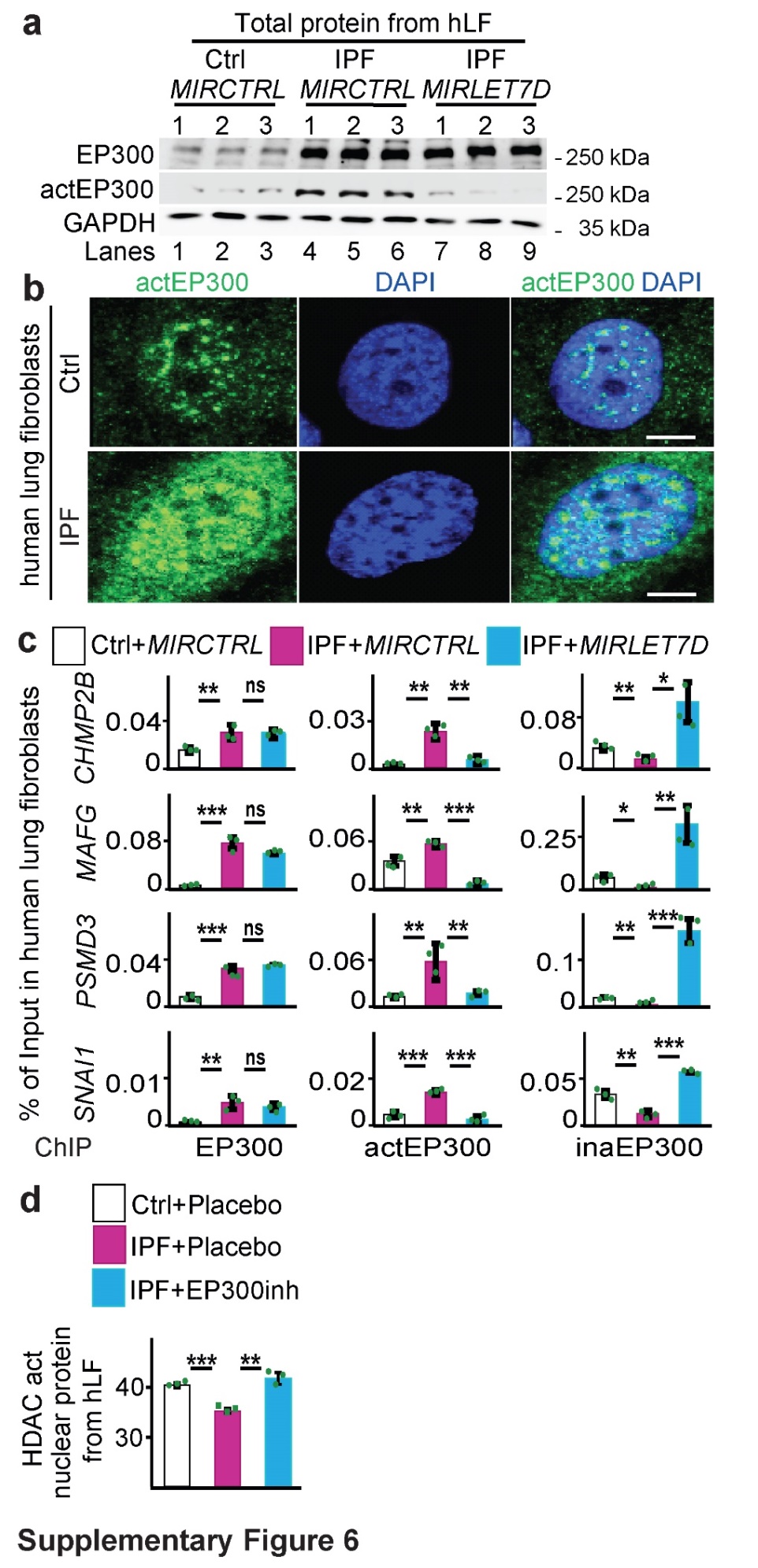


**Supplementary Figure 6.**

**Accumulation of active EP300 in IPF fibroblasts is inhibited by *MIRLET7D*.** **a**, WB using the indicated antibodies and whole-cell protein extracts from Ctrl and IPF primary human lung fibroblasts (hLF) derived from three different donors, each, and transfected with *MIRCTRL* or *MIRLET7D*. See Figure 6. **b**, Confocal microscopy after actEP300-immunostaining shows its increased nuclear localization in IPF but not in IPF primary fibroblasts. ActEP300, active EP300. DAPI, nucleus. Scale bars, 10 μm. **c**, ChIP analysis of representative *MIRLET7D* target promoters using EP300, actEP300 or inaEP300-specific antibodies and chromatin isolated from Ctrl or IPF primary fibroblasts that were transfected with control miRNA (*MIRCTRL)* or *MIRLET7D* probes. IgG, immunoglobulin G (negative control). ActEP300, active EP300. **d**, HDAC activity assay using nuclear protein extracts from Ctrl or IPF hLF that were treated with placebo (vehicle solution) or 10 µM EP300 inhibitor (EP300ihn, CBP30). See Figure 5b. In all bar plots, data are shown as mean (SD) (*n*=3 biologically independent experiments). Asterisks in all plots, *P* values after unpaired t-Test, two tailed, ****P*˂0.001; ***P*˂0.01; **P*˂0.05. Source data are provided as a Source Data file.


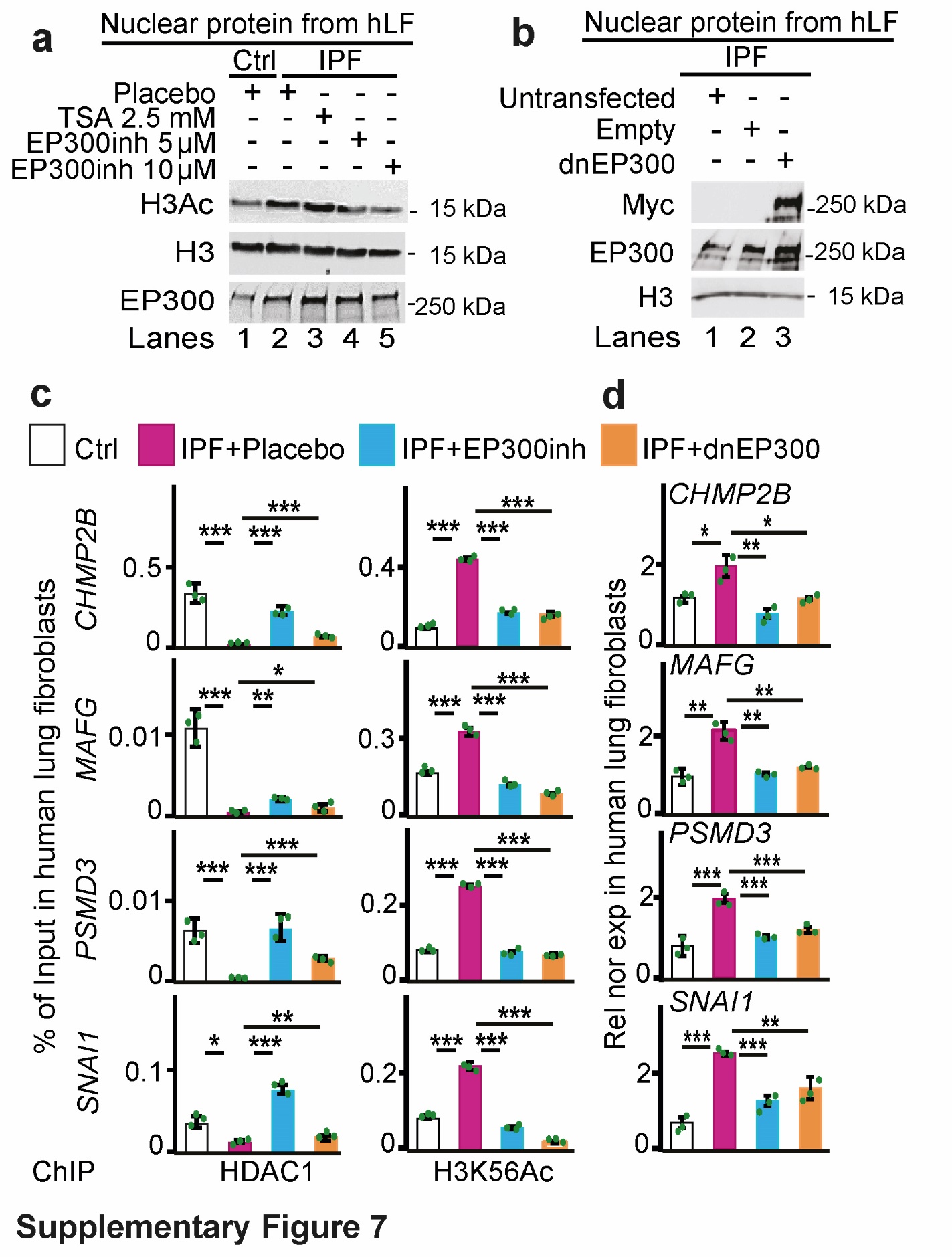


**Supplementary Figure 7.**

**EP300 inhibition re-activates nuclear HDAC1 in IPF.** **a**, WB using the indicated antibodies and whole-cell protein extracts from Ctrl and IPF primary fibroblasts that were treated with TSA or with 2 different concentrations of EP300 inhibitor (EP300inh, 5 μM or 10 μM) showed decreased H3 acetylation upon EP300inh treatment. **b**, WB using the indicated antibodies and whole-cell protein extracts from Ctrl and IPF primary fibroblasts that were transfected with a MYC-tagged dominant negative EP300 (dnEP300) construct using MYC, EP300 and H3-specific antibodies. **c**, ChIP analysis of representative *MIRLET7D* target promoters using HDAC1 or H3K56Ac-specific antibodies and chromatin isolated from Ctrl or IPF primary fibroblasts after treatment with EP300inh or transfection of a dominant negative EP300 (dnEP300) construct. IgG, immunoglobulin G (negative control). **d**, qRT-PCR-based analysis of cRNA of representative nuclear *MIRLET7D* targets in Ctrl and IPF primary fibroblasts treated with EP300inh or overexpressing a *dnEP300* construct. In all bar plots, data are shown as mean (SD) (*n*=3 biologically independent experiments). Asterisks in all plots, *P* values after unpaired t-Test, two tailed, ****P*˂0.001; ***P*˂0.01; **P*˂0.05; ns, not significant. Source data are provided as a Source Data file.


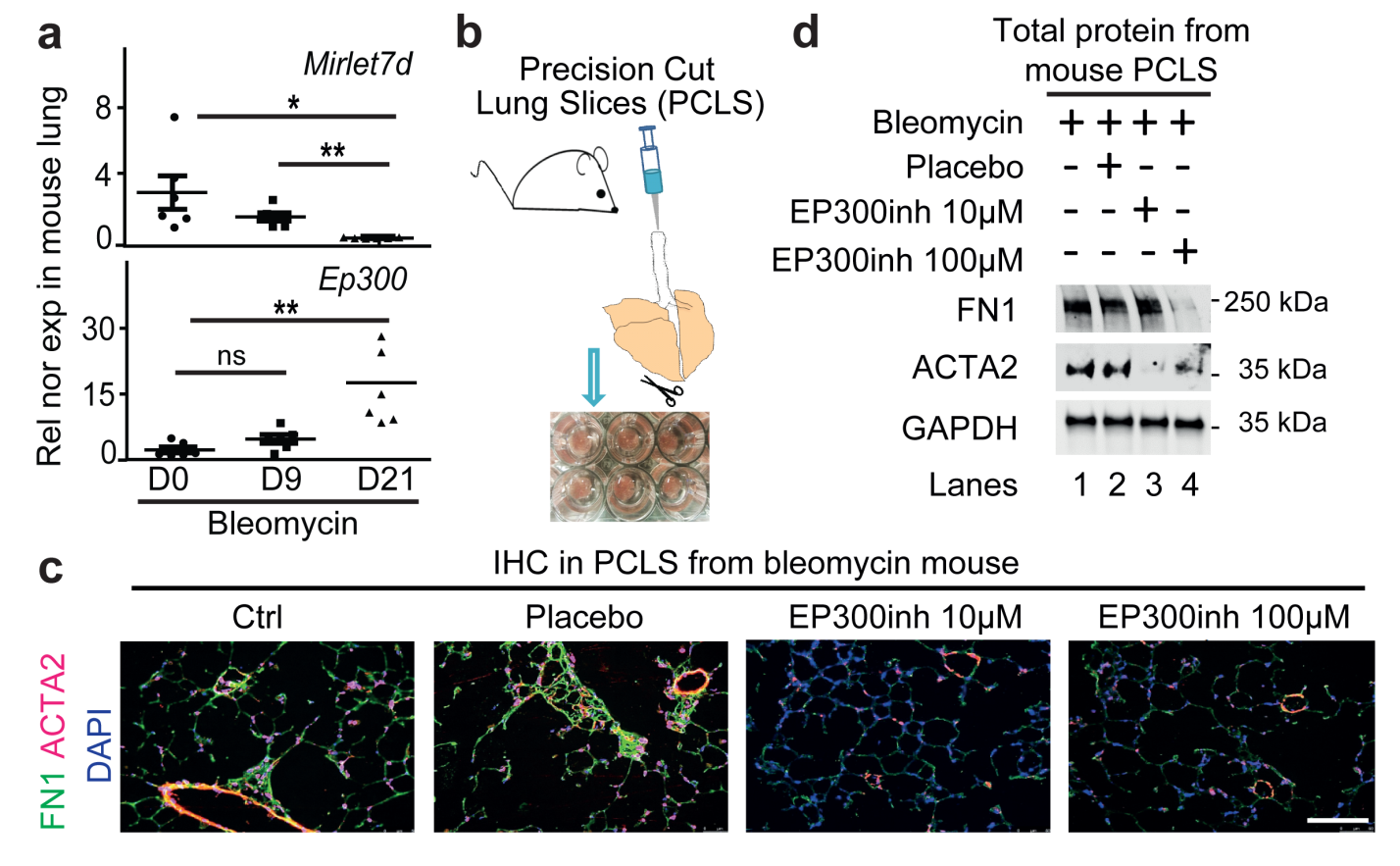


**Supplementary Figure 8.**

**EP300 inhibition counteracts fibrotic hallmarks in IPF.** **a**, Mature *MIRLET7D*-specific TaqMan assay and qRT-PCR analysis of *Ep300* using total lung homogenates from D0, D9 and D21 (*n*=6 mice, each) bleomycin-treated mice. Rel norm exp, relative normalized expression. **b**, Scheme shows PCLS experimental setup. Mice were orotracheally instilled with bleomycin (2.5 U/kg body weight) at day 0. PCLS were prepared at day 21 post-instillation and subsequently *ex vivo* treated with placebo (vehicle solution) or EP300 inhibitor (EP300inh, CBP30 10μM or 100μM) on days 1 and 2. PCLS were harvested for analysis at day 3. **c**, Confocal microscopy after co-immunostaining using FN1 and ACTA2-specific antibodies in PCLS treated with placebo (vehicle solution) or two different concentrations of EP300 inhibitor (EP300inh, CBP30, 10 μM or 100 μM). DAPI, nuclear staining. Scale bar, 500 µm. **d**, WB using the indicated antibodies and whole-cell protein extracts from representative bleomycin-treated mice PCLS treated with placebo (vehicle solution) or two different concentrations of EP300 inhibitor (EP300inh, CBP30, 10 μM or 100 μM). Data are shown as mean (SD) (*n*=6 biologically independent animals). Asterisks in all plots, P values after unpaired t-Test, two tailed, ***P˂0.001; ns, not significant. Source data are provided as a Source Data file.


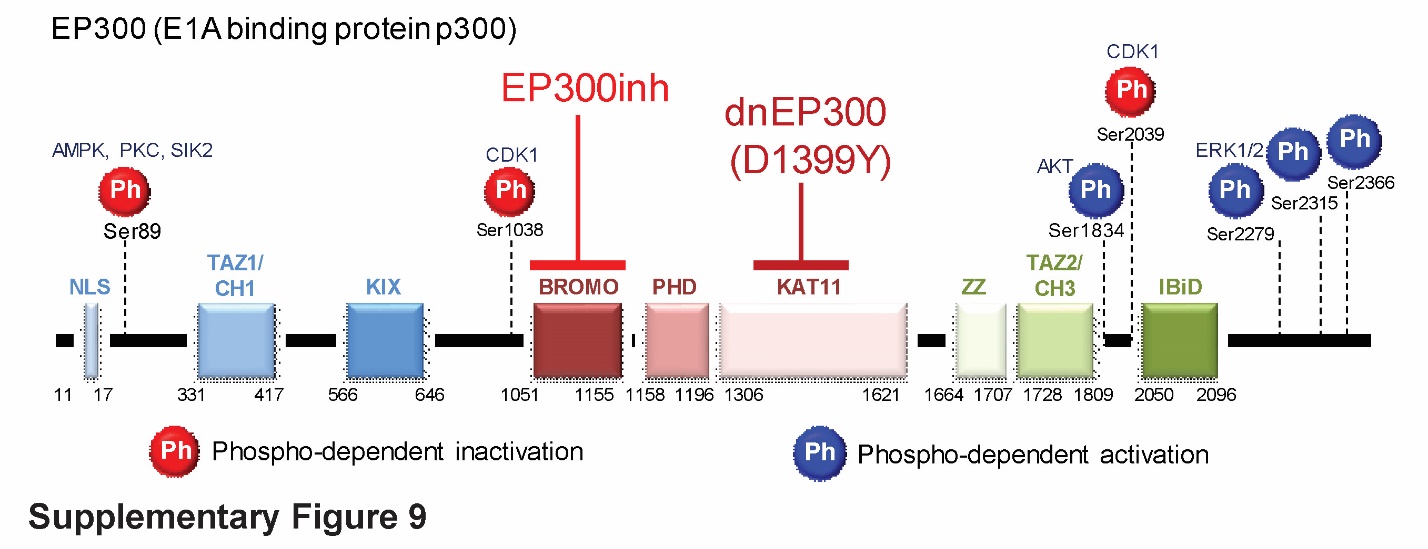


**Supplementary Figure 9.**

**Domain structure and phosphorylation residues of EP300.** EP300 (E1A binding protein p300) shares a tri-modular organization consisting of an N-terminal transactivation domain (blue), a central chromatin association and modification region (red) and a C-terminal transactivation domain (green). Functional domains of EP300 are nuclear localization signal (NLS), cysteine ͞histidine-rich domains (CH1 and CH3), CREB-binding domain (KIX), bromodomain (BROMO), histone acetyltransferase domain (KAT11), transactivation domain (ZZ) and IRF-3-binding domain (IBiD). The CH1 and CH3 domains are structurally homologous and have been termed TAZ1 and TAZ2, respectively. Serine residues important for EP300 phospho-dependent activation are depicted in circles (red, inactivation; blue, activation). The bromodomain/PHD finger module and the KAT11 domain are flanked by four transactivation modules: TAZ1/CH1, KIX, TAZ2/CH3/ZZ and IBiD. The bromodomain mediates EP300 binding to acetylated histones, nucleosomes and transcriptional factors that tether EP300 to specific chromosomal sites. Active EP300 (actEP300) is detected with antibodies specific for Phospho-EP300 Ser1834 residue, while inactive EP300 (inaEP300) is detected with antibodies specific for Phospho-EP300 Ser89 residue.

**Supplementary Table 1.**

List of Oligonucleotides

| **Gene** | **Fwd Primer Sequence (5’-3’)** | **Rwd Primer Sequence (5’-3’)** |
| --- | --- | --- |
| *hCHMP2B (cRNA)* | 5‘-ACAATGCAGGCAGTTAACAAGA-3‘ | 5‘-TCCTGGCTTTCTTCTTCGTC-3‘ |
| *hMAFG (cRNA)* | 5‘-TGACGACCCCCAATAAAGGA-3‘ | 5‘-CGATCTCCTCCTTGGACAGG-3‘ |
| *hNOLC1 (cRNA)* | 5‘-GCATCATCCCCATTCCGAAGG-3‘ | 5‘-ACCTGATTGGCTCGCTCTCC-3‘ |
| *hPSMD3 (cRNA)* | 5‘-GTGCAGGGCTTCTTCACTTC-3‘ | 5‘-ATGACCACGAGGAGTTGGAG-3‘ |
| *hSNAI1 (cRNA)* | 5‘-GTGCCTCGACCACTATGCCG-3‘ | 5‘-GGGCTGCTGGAAGGTAAACTCTGG-3‘ |
| *hHPRT (cRNA)* | 5‘-TTTGCTTTCCTTGGTCAGGCAGT-3‘ | 5‘-CGTGGGGTCCTTTTCACCAGCA-3‘ |
| *hGAPDH (cRNA)* | 5‘-GGCCCGATTTCTCCTCCGGGT-3‘ | 5‘-GGTGACCAGGCGCCCAATACG-3‘ |
| *hMAFG (*ChIP) | 5‘-TGGTTGTTAAGTCCGGCCAC-3‘ | 5‘-CCTCTAGACCTCCCAGTCGG-3‘ |
| *hPSMD3* (ChIP) | 5‘-CAATGGGCTCCCTAAAGCGT-3‘ | 5‘-GGGAGTGCCGATAGGTAGGA-3‘ |
| *hCHMP2B (ncRNA)* | 5‘-TGAACATTATTCCAGATTTAATGGC-3‘ | 5‘-CAGAAGGCAAACTCAGTGAACA-3‘ |
| *hMAFG (ncRNA)* | 5‘-GGCTGCGTGTGGTTCTTAC-3‘ | 5‘-CCCCGTGTGCTGTTTTGTAC-3‘ |
| *hNOLC1 (ncRNA)* | 5‘- GCTCAGTGCTGGTAATGGTGTA -3‘ | 5‘- CCACCTCCTTCACATCGAGTCTC-3‘ |
| *hPSMD3 (ncRNA)* | 5‘-GCAGACATAAGCAAACCCAGT-3‘ | 5‘-ACTGACCAAGATCCAGAGGG-3‘ |
| *hCHMP2B* (ChIP) | 5‘-CGGGGATCCAGTGTCTAGAG-3‘ | 5‘-TCACCGTATAGCCATGACAC-3‘ |
| *hNOLC1* (ChIP) | 5‘-TTTCACTGTGTTGTCCAGGC-3‘ | 5‘-TACCTCCCACTAATGCCGC-3‘ |
| *hSNA1I* (ChIP) | 5‘-AGCACCGGGGACGACCCG-3‘ | 5‘-GGCCTTATCTGCCACGCCCCTTT-3‘ |
| *mEp300 (cRNA)* | 5’-AATGGCCGAGAATGTGGTGGAACC-3’ | 5’-GCTGACTGATATCGCCACCATTGGT-3’ |
| *mGapdh (cRNA)* | 5’- TGAGTATGTCGTGGAGTCTAC -3’ | 5’- TGGACTGTGGTCATGAGCC-3’ |

**Source Data File (separate file)**

Excel file containing the values and statistical summary of the complete manuscript, and theuncropped pictures of all the western blots presented in the manuscript.

Supplementary References

1. Dovey OM, Foster CT, Cowley SM. Histone deacetylase 1 (HDAC1), but not HDAC2, controls embryonic stem cell differentiation. *Proceedings of the National Academy of Sciences of the United States of America* **107**, 8242-8247 (2010).

2. Luo Y*, et al.* Trans-regulation of histone deacetylase activities through acetylation. *The Journal of biological chemistry* **284**, 34901-34910 (2009).

3. Singh I*, et al.* MiCEE is a ncRNA-protein complex that mediates epigenetic silencing and nucleolar organization. *Nature genetics*, (2018).
