# Supplementary material for: Inactivation of nuclear histone deacetylases by EP300 disrupts the MiCEE complex in idiopathic pulmonary fibrosis
Source: Nat Commun. 2019 May 20;10:2229. doi: 10.1038/s41467-019-10066-7 (PMC6527704; doi:10.1038/s41467-019-10066-7)
Supplement: Supplementary file 2 — Reporting Summary [file 41467_2019_10066_MOESM2_ESM.pdf]

nature research

Corresponding author(s): Guillermo Barreto, Indrabahadur Singh

Last updated by author(s): Feb 25, 2019

Reporting Summary

Nature Research wishes to improve the reproducibility of the work that we publish. This form provides structure for consistency and transparency in reporting. For further information on Nature Research policies, see Authors & Referees and the Editorial Policy Checklist.

Statistics

For all statistical analyses, confirm that the following items are present in the figure legend, table legend, main text, or Methods section.

n/a	Confirmed	
☐	☒	The exact sample size (*n*) for each experimental group/condition, given as a discrete number and unit of measurement
☐	☒	A statement on whether measurements were taken from distinct samples or whether the same sample was measured repeatedly
☐	☒	The statistical test(s) used AND whether they are one- or two-sided<br>*Only common tests should be described solely by name; describe more complex techniques in the Methods section.*
☐	☒	A description of all covariates tested
☐	☒	A description of any assumptions or corrections, such as tests of normality and adjustment for multiple comparisons
☐	☒	A full description of the statistical parameters including central tendency (e.g. means) or other basic estimates (e.g. regression coefficient) AND variation (e.g. standard deviation) or associated estimates of uncertainty (e.g. confidence intervals)
☐	☒	For null hypothesis testing, the test statistic (e.g. *F*, *t*, *r*) with confidence intervals, effect sizes, degrees of freedom and *P* value noted<br>*Give P values as exact values whenever suitable.*
☒	☐	For Bayesian analysis, information on the choice of priors and Markov chain Monte Carlo settings
☒	☐	For hierarchical and complex designs, identification of the appropriate level for tests and full reporting of outcomes
☐	☒	Estimates of effect sizes (e.g. Cohen's *d*, Pearson's *r*), indicating how they were calculated

Our web collection on statistics for biologists contains articles on many of the points above.

Software and code

Policy information about availability of computer code

Data collection	Excel solver, R, GraphPrism (v. 5), FastQC, trimmomatic, Bowtie2, BWAm, samtools view (-Sb), Samtools sort, picard-tools-1.119, HOMER, KNIME 2.9.1, RNAhybrid, UCSC genome browser, DAVID
Data analysis	Excel solver and R-scripts were used to analyze the statistics. Bar graph was made using GraphPrism (v. 5). The box-and-whisker plots were created with a custom made R-script. Sequencing raw reads were visualized by FastQC. Low quality reads were filtered out by using trimmomatic. Bowtie2 or BWA was used for mapping of trimmed sequencing reads. The sam files were converted to bam format by using samtools view (-Sb) followed by Samtools sort. The bam files were converted to fastq files with SamToFastq.jar from picard-tools-1.119. The tag libraries were created and samples were quantified using HOMER. KNIME 2.9.1 software was used for crossing and analyzing more than one data sets. RNAhybrid-online server was used for miRNA binding sites prediction. UCSC genome browser was used for visualization of gene loci. DAVID gene functional classification tool was used for gene ontology analysis.

For manuscripts utilizing custom algorithms or software that are central to the research but not yet described in published literature, software must be made available to editors/reviewers. We strongly encourage code deposition in a community repository (e.g. GitHub). See the Nature Research guidelines for submitting code & software for further information.

Data

Policy information about availability of data

All manuscripts must include a data availability statement. This statement should provide the following information, where applicable:

- Accession codes, unique identifiers, or web links for publicly available datasets
- A list of figures that have associated raw data
- A description of any restrictions on data availability

Sequencing data nuclear RNA in Ctrl and IPF patients have been deposited in NCBI's Gene Expression Omnibus 50 and is accessible through GEO Series with accession number GSE116086. RNA-seq data from total lung homogenates was retrieved through the GEO Series accession number GSE52463.

Field-specific reporting

Please select the one below that is the best fit for your research. If you are not sure, read the appropriate sections before making your selection.

☒ Life sciences ☐ Behavioural & social sciences ☐ Ecological, evolutionary & environmental sciences

For a reference copy of the document with all sections, see nature.com/documents/nr-reporting-summary-flat.pdf

Life sciences study design

All studies must disclose on these points even when the disclosure is negative.

Sample size	No statistical method was used to pre-select the sample size. We performed each experiment 3 times independent from each other, unless it is indicated differently. In each experiment the samples were measured at least in duplicate. Depending on the data, different tests were performed to determine the statistical significance of the results. One set of nuclear RNA was analyzed by deep sequencing using biological replicates. The values of the statistical tests used in the different experiments can be found in the Source data files.
Data exclusions	No data were excluded from the analyses.
Replication	For each experiment, all attempts at replication were successful.
Randomization	No method of randomization was used. Experiments were performed 3 times independent from each other, unless it is indicated differently. In each experiment the equivalent samples were treated as equally as possible. Statistical relevant results were reproducible.
Blinding	Investigators were not blinded during the experiments. Experiments were performed 3 times independent from each other, unless it is indicated differently. In some cases, the experiments were performed by different researchers providing reproducible and statistical relevant results.

Reporting for specific materials, systems and methods

We require information from authors about some types of materials, experimental systems and methods used in many studies. Here, indicate whether each material, system or method listed is relevant to your study. If you are not sure if a list item applies to your research, read the appropriate section before selecting a response.

Materials & experimental systems

n/a	Involved in the study	
☐	☒	Antibodies
☒	☐	Eukaryotic cell lines
☒	☐	Palaeontology
☐	☒	Animals and other organisms
☐	☒	Human research participants
☒	☐	Clinical data

Methods

n/a	Involved in the study	
☒	☐	ChIP-seq
☒	☐	Flow cytometry
☒	☐	MRI-based neuroimaging

Antibodies

Antibodies used	Reported in Material and Methods section. Primary antibodies used in this study are anti-EXOSC10 (Santa Cruz # sc-374595), anti-EZH2 (Abcam, # ab3748), anti-H3K27me3 (Diagenode, # C15410069 (pAb-069-050)), anti-H3K27me3 (Millipore, # 07-449), anti-H3 (Abcam, #1791), anti-C1D (Aviva, # ARP50534_P050), anti-EXOSC10 (Abcam, # ab50558), anti-EXOSC5 (Abcam, # ab69699), anti-EXOSC1 (Abcam, #181167), anti-CTNNB (Abcam, #7302), anti-SUV39H1 (Active Motif, #39785),

	anti-H3K56ac (Epigentek, #A-4026-050), anti-H3K27ac (Abcam, #ab4729), anti-H3Ac (Abcam, #ab47915), anti-FN1 (Millipore, #AB2033), anti-COL1A1 (Sigma, #C2456), anti-ACTA2 (Sigma, #A5228), anti-GAPDH (Sigma, #G8795), anti-HA (Santa Cruz, #sc-805), anti-MYC (Abcam, #9132)), anti-HDAC1 (Abcam, #ab7028), anti-HDAC2 (Abcam, #ab16032), anti-POLII (Abcam, #ab5408), anti-EP300 (Abcam, #59240), anti-actEP300 (Thermo Fisher, PA5-64531), anti-actEP300 (Biorbyt, #34557), anti-inaEP300 (Santa Cruz, #130210), anti-AcK (Cell Signaling, #9441S)), anti-PhS (Millipore, #A-570-05-1000X), anti-CTNNB (Abcam, #ab6302), Anti-FLAG M2 (Sigma, #F1804), anti-LMNB1 (Santa Cruz, # sc-6216), anti-ACTB (Santa Cruz, #sc-1616), anti-GAPDH (Sigma, # G8795), anti-IgG (Santa Cruz, # sc-2027) and anti-IgG (Santa Cruz, # sc-2025).
Validation	Species validation of all primary antibodies used in this study can be found in the corresponding manufacturer's websites.

Animals and other organisms

Policy information about studies involving animals; ARRIVE guidelines recommended for reporting animal research

Laboratory animals	8- to 10-week-old male mice were orotracheally instilled with 2.5 U/kg body weight of bleomycin (Medac, Germany) that was dissolved in saline. Control groups were instilled with saline. Bleomycin-instilled mice were orotracheally instilled with Placebo or EP300inh (11 nmol/20g body weight) on d9, d12, d15 and d18.
Wild animals	Wild animals were not used in this work.
Field-collected samples	Field-collected samples were not used in this work.
Ethics oversight	Animal experiments were performed in accordance with German animal protection laws and were approved by the local governmental animal protection committee (approval number B2/1165).

Note that full information on the approval of the study protocol must also be provided in the manuscript.

Human research participants

Policy information about studies involving human research participants

Population characteristics	Patient clinical characteristics are summarized in the Figure S1b.
Recruitment	All patient and control materials were obtained through the European IPF Registry (www.pulmonary-fibrosis.net), the UGMLC Giessen Biobank (member of the DZL Platform Biobanking) and the Biobank from the Institute for Pathology of the Hannover Medical School as part of the BREATH Research Network. We used anonymized patient material.
Ethics oversight	This study was performed according to the principles set out in the WMA Declaration of Helsinki; the underlying protocols were approved by the ethics committee of Medicine Faculty of the Justus Liebig University in Giessen, Germany (AZ.111/08-eurIPFreg) and the Hannover Medical School (no. 2701-2015).

Note that full information on the approval of the study protocol must also be provided in the manuscript.
